# Longitudinal Effects of a Smartphone Game (Tumaini) for HIV Prevention Among Kenyan Adolescents: 45-Month Trajectories of Condom Use–Related Proximal Outcomes From a Randomized Controlled Trial

**DOI:** 10.2196/83982

**Published:** 2026-03-10

**Authors:** Kate Winskell, Gaëlle Sabben, Haowen Qin, Calvin Mbeda, Sophie Goldenberg, Ken Ondeng'e, Richard Ndivo, Judith Arego, Xinwei He, Robert A Bednarczyk, Kelli Komro, Robert H Lyles, Victor Mudhune

**Affiliations:** 1 Hubert Department of Global Health Rollins School of Public Health at Emory University Atlanta, GA United States; 2 Department of Biostatistics and Bioinformatics Rollins School of Public Health at Emory University Atlanta, GA United States; 3 HIV Research Division Centre for Global Health Research Kenya Medical Research Institute Kisumu Kenya; 4 Department of Epidemiology Rollins School of Public Health at Emory University Atlanta, GA United States; 5 Department of Behavioral, Social, and Health Education Sciences Rollins School of Public Health at Emory University Atlanta, GA United States

**Keywords:** adolescents, HIV, prevention, sexual health, sub-Saharan Africa, Kenya, behavioral intervention, serious game, game for health, interactive narrative, smartphone, randomized controlled trial, mHealth, condom, behavioral intention, self-efficacy, attitude, knowledge

## Abstract

**Background:**

African adolescents and young adults account for a disproportionate number of new HIV infections. There is an urgent need to identify scalable and cost-effective behavioral HIV prevention strategies for this population. Using a condom at first sex is associated with a higher likelihood of consistent use later. Tumaini (“Hope for the Future” in Swahili; Emory University) is a choose-your-own-adventure smartphone game that has been shown to reduce the risk of unprotected first sex by end line in a 45-month randomized controlled trial in western Kenya.

**Objective:**

This study aimed to assess the impact of Tumaini on proximal outcomes related to condom use at first sex (specifically, behavioral intentions, self-efficacy, attitudes, and knowledge) longitudinally across mid-adolescence in the above trial.

**Methods:**

Adolescent participants (n=996, mean baseline age 14, SD 0.56 years) were randomized 1:1 to receive either a smartphone loaded with Tumaini or an attention-control math game for 5 to 7 weeks at 3 time points (mean age 14.0, SD 0.56; 15.3, SD 0.55; and 16.0, SD 0.56 years, respectively). They completed a behavioral survey at 13 time points, through mean age 17.7 (SD 0.56) years. Using generalized estimating equations and controlling for age at baseline, we modeled mean scores (overall and stratified by gender) on a range of condom-related survey items over time to assess mean differences at specific time points. We applied appropriate Bonferroni corrections to inferences about cross-arm differences in mean changes relative to baseline at 4 time points (after each intervention period and at end line; α=.05/4) and within-arm mean changes relative to baseline at each of the 12 post-baseline time points (α=.05/12). Analyses were conducted as intent-to-treat.

**Results:**

At end line, 97.8% (n=974) of the sample had been retained. Participants in both arms dedicated a mean total of >30 hours to their assigned game. There was significant improvement across all condom-related proximal outcomes in the intervention arm relative to the control arm immediately after initial intervention exposure. For almost all outcomes, a significant cross-arm difference was also present at end line and for most outcomes at the 2 intervening comparison time points. Some outcomes saw stronger intervention effects on female participants (eg, self-efficacy to refuse unprotected sex) or male participants (eg, knowledge that condoms are an effective way to prevent HIV). In each arm, intention to use a condom at first sex was consistently higher among male participants; however, female intervention-arm scores overtook male control-arm scores following initial intervention exposure.

**Conclusions:**

Tumaini significantly improved theory-based proximal outcomes related to condom use, with effects sustained 45 months post initial exposure and 16 months post most recent exposure. Adolescents benefited from even short-term exposure, though repeated exposure generally sustained and reinforced intervention effects. As access to smartphones increases, Tumaini has potential for high scalability and impact on condom-related outcomes.

**Trial Registration:**

ClinicalTrials.gov NCT04437667; https://clinicaltrials.gov/study/NCT04437667

**International Registered Report Identifier (IRRID):**

RR2-10.2196/35117

## Introduction

Worldwide, a third of all new adult (aged 15-49 years) HIV infections occur in eastern and southern Africa, and over a third of these infections occur in young people aged 15-24 years [1]. HIV-related morbidity and mortality in this age group are disproportionately high. In addition, as the largest-ever generation of young Africans is now entering adolescence, it is feared that a resurgence in the global epidemic could ensue unless more is done to prevent HIV in this demographic [2]. There is an urgent need to develop and rigorously test scalable and cost-effective behavioral interventions to prevent HIV among African adolescents and young adults, particularly adolescent girls and young women who are disproportionately affected [1].

Reaching adolescents with information and skills before sexual debut may help establish long-term patterns of safer sexual behavior [3]. For example, studies have shown that those who use condoms at first sex are more likely to use them consistently in the future [4]. There is a need for a stronger evidence base on the longitudinal effects of early and ongoing intervention, as few studies evaluating any HIV prevention interventions for adolescents and young adults include long-term follow-up [5].

Scaling up in-person, group-based HIV prevention interventions is challenging [6]. Digital behavioral interventions are likely to be easier to deliver at scale with consistent quality than those delivered in person. As smartphone penetration grows across sub-Saharan Africa, especially among young people, digital HIV prevention interventions are becoming increasingly viable. Across the continent, 81% of mobile phone connections will be smartphones by 2030 [7].

Electronic games are a promising subset of digital behavioral interventions [8]. However, few have been rigorously designed and evaluated [9]. Specifically, games leveraging interactive narrative can provide a level of experiential learning unparalleled by most other interventions by allowing players to experience real agency in a virtual and safe environment.

Tumaini (“hope for the future” in Swahili) is a “choose-your-own-adventure” game app for inexpensive Android smartphones. It was developed by specialists in adolescent sexual health (KW, GS, with others) in collaboration with a commercial game developer with input from Kenyan adolescents and their parents. The game’s theoretical framework [10] draws on Social Cognitive Theory [11], existing evidence-based interventions for youth HIV prevention, and literature on games for health and Entertainment-Education [12,13]. It is grounded in research on HIV-themed narratives written by thousands of young Africans [14-16].

Tumaini uses interactive narrative to promote observational learning, cognitive and behavioral rehearsal, and problem-solving [10]. It provides over 12 hours of discrete gameplay and comprises (1) an engaging role-playing narrative in which players make choices for 6 characters (3 female, 3 male) as they proceed through adolescence and observe short- and long-term consequences in the characters’ lives; (2) mini-games to reinforce knowledge and skills; and (3) My Story, in which players set life goals, reflect on how the game relates to their lives, customize a personal avatar, and collect rewards. The narrative uses branching logic for 40 possible long-term outcomes (“epilogues”) across the 6 characters and is designed to be replayed, allowing players to experience different outcomes based on their in-game decisions. Players must assume the role of characters different from themselves in terms of sex and serostatus, thereby challenging stigma and harmful gender norms.

In a randomized feasibility study with 60 adolescents in Kenya, the intervention arm showed significant gains in sexual health-related knowledge and self-efficacy and behavioral intention for risk avoidance strategies and sexual risk communication when compared with the control arm 6 weeks postintervention [17]. Intervention-arm participants spent on average over 50% longer playing the game than instructed, and quantitative and qualitative data on user engagement and on game appeal, relevance, and acceptability among adolescents and parents were extremely positive [18].

A randomized controlled trial with a larger sample size (n=996) and longer follow-up (45 months) was undertaken with the objective of determining the intervention’s effects on (1) age at sexual debut and condom use at first sex and (2) related proximal outcomes (behavioral intentions, self-efficacy, attitudes, and knowledge). Tumaini demonstrated significant impact on the primary trial outcome (high-risk sexual debut vs low-risk or no sexual debut), attributable to increased condom use at first sex, at end line in intent-to-treat analyses [19]. Significant effects were also observed across a wide range of other outcomes at the end line.

Participants completed a behavioral survey at 13 time points over the 45-month duration of the trial, with a mean participant age of 14.0 to 17.7 (SD 0.56) years. The survey included items measuring a range of theory-based proximal outcomes related to condom use. These data and their longitudinal trajectories are the focus of the analyses presented in this manuscript. As the primary behavioral outcomes are related to sexual debut and developmentally influenced, hence statistically underpowered prior to end line, measures of proximal outcomes are of particular importance to understanding the effects of the intervention between trial baseline and end line. The purpose of this manuscript is to leverage these longitudinal data to evaluate Tumaini’s impact on proximal outcomes related to condom use across 45 months of mid-adolescence, a key developmental phase, while assessing differential effects by gender.

## Methods

### Study Setting

Kenya has the 7th largest HIV burden globally and an HIV epidemic that is generalized throughout the population. Kisumu County has Kenya’s second-highest prevalence (15.5%) and annual number of new infections [20]. The study took place in Kisumu Town, Kenya’s third largest city, where youth aged 15-24 years comprise 48% of new HIV infections [21].

### Study Design

This was a 45-month, 2-arm, individually randomized controlled trial with 996 participants, with a mean age of 14.0 (SD 0.6) years at baseline [22]. The study took place from October 2020 (start of enrollment) to August 2024 (last quantitative data collection). Participants were randomized 1:1 to play either Tumaini or a commercially available attention-control math game, Brainilis (Appgeneration – Software Technologies, Lda)*.* They were provided with a low-cost Android smartphone loaded with their assigned game at mean participant age 14.0 (SD 0.56), 15.3 (SD 0.55), and 16.0 (SD 0.56) years, that is, between baseline data collection (T1) and second data collection time point (T2), 6th data collection time point (T6) and 7th data collection time point (T7), and 8th data collection time point (T8) and 9th data collection time point (T9). Gameplay periods lasted 5-7 weeks and took place during school holidays; they were unevenly spaced due to COVID-19–related disruptions to the school calendar. Participants completed behavioral surveys at 13 time points (baseline plus 12 follow-up surveys).

### Recruitment and Randomization

Participant recruitment was conducted through a combination of school- and community-based strategies in urban and periurban Kisumu. Eligibility criteria for participation were being aged 12-14 years at the time of enrollment, being a resident in Kisumu Town, having basic English literacy (Grade 3-4 on the Flesch-Kincaid Reading Scale), and having no prior exposure to Tumaini-related activities.

Recruitment slots were used to balance participant gender, age, and residence (in the 16 wards that comprise Kisumu East, West, and Central). Randomization to the study arms was conducted by a blinded member of the data analysis team with no contact with participants. The success of randomization was confirmed through calculation and cross-arm comparison of descriptive statistics for adolescent demographics.

Due to the nature of the intervention, participants were not blinded to group assignment, which was revealed following completion of all baseline activities. Data analysts remained blinded to study arm assignment until completion of all primary outcome data analysis.

### Data Collection and Analysis

Behavioral surveys were self-administered at 13 time points (T1-T13; 13th data collection time point [endline]), via tablet-based Open Data Kit. Participants completed surveys with headphones in a private space. Access to audio of all questions was provided to maximize understandability. Survey items were designed to evaluate the impact of exposure to the intervention on a range of primary and secondary outcomes. The study’s primary outcomes assessed experience of sexual debut and condom use at first sex separately and also combined them in a composite binary variable of high-risk sexual debut (no condom use or “I don’t know”) vs low-risk sexual debut (condom use) or no risk (not yet initiated sex). Sex was defined as penile-vaginal (“when a boy or man puts his penis inside a girl’s or woman’s vagina”). It was specified that condoms were understood to be male condoms, defined as “a thin rubber covering worn by a man on his erect penis when he has sex,” due to the low availability of female condoms (or “internal condoms”). Endline outcome analyses are presented elsewhere [19].

Survey items also assessed theory-based and intervention-targeted proximal influences, namely knowledge, attitudes, self-efficacy, and behavioral intentions, of these primary outcomes. The behavioral survey instrument was adapted from the version used in the Tumaini feasibility study [17]. Individual measures were drawn from existing instruments, with priority given to those previously used with sub-Saharan African youth populations [23-25]. The chosen items were adapted for age, linguistic, and cultural appropriateness and consistency of formatting, and supplemented with additional measures where necessary. In the interest of age appropriateness, some questions were framed as hypothetical risk scenarios (“*Imagine that*…”).

Here, we focus on the longitudinal trajectories of the proximal outcomes related to condom-use behavior. These outcomes, their exact wording, and response options (binary and 3 or 4-point Likert scales) are presented in Table 1 (additional items are detailed in Table S1 in Multimedia Appendix 1). Each item was scored on a 0-1 scale, with higher values indicating more desirable or, in the case of knowledge, correct answers. This standardization of scores facilitated comparison across items. Mean scores were calculated for individual items at each time point. Only participants who had responded to the question at that time point were included in mean score calculations; missing values were not imputed. Only those participants reporting never having had sex were asked about their intention to use a condom at first sex.

**Table 1:** Condom-related proximal outcomes, behavioral survey question wording, and response options for Tumaini efficacy trial.

Condom-related proximal outcome			Question wording		Response options
**Behavioral intention**					
	Intention to use a condom at first sex	“When you have sex for the first time, do you plan to use a condom?”		Definitely yes, maybe yes, maybe no, definitely no	
	Intention to talk to a partner about HIV prevention	“Imagine that either in the future or now you are thinking about having sex with someone and you are not ready to become a parent. […] Would you talk with him/her about preventing HIV?”		Definitely yes, maybe yes, maybe no, definitely no	
**Self-efficacy**					
	Self-efficacy to use a condom correctly	“I am sure that I could use a condom correctly.”		I strongly agree, I agree, I disagree, I strongly agree	
	Self-efficacy to refuse unprotected sex	“Imagine that, either in the future or now, you are in a relationship. He/ she says she wants to have sex with you, but she does not want to use a condom. […] If I did not want to have sex with him/her without a condom, I am sure that I could say no firmly.”		I strongly agree, I agree, I disagree, I strongly agree	
**Attitudes**					
	Endorsement that condom use is a sign of respect	“Using a condom is a sign that you respect your partner”		I strongly agree, I agree, I disagree, I strongly agree	
	Endorsement of gender parity in condom initiation	“A woman can suggest using condoms just like a man can”		I strongly agree, I agree, I disagree, I strongly agree	
**Knowledge**					
	Knowledge of how to use a condom correctly	“Do you know how to use a condom correctly?”		Yes, no	
	Knowledge that condoms are effective for HIV prevention	“Are condoms an effective way to prevent HIV?”		Yes, no, I don’t know	

We used generalized estimating equations [26] to model these proximal outcome variables measured repeatedly over time and to facilitate statistical inferences about cross-arm (intervention vs control) mean differences at specific time points. Specifically, the generalized estimating equations model controlled for participants’ baseline age (centered at the average value; due to the success of randomization, we did not control for any other covariates) and included indicator variables for arm (intervention as reference) and time (T1–T13, with T1 as reference), together with the 12 group-by-time interaction terms. This approach accounts for the correlated nature of the data, using an exchangeable working correlation structure that was selected via initial comparisons of alternatives based on the quasi-likelihood information criterion [27]. The granular nature of the model provides a close fit to the sample means of the outcome for each arm at each time, while facilitating direct inferences about the change in the mean outcome relative to baseline for the intervention arm at each time point (T2–T13) and the cross-arm differences in these mean changes. Of primary interest in these analyses are Bonferroni-corrected (significance level .05/4) inferences about the cross-arm differences in mean changes relative to baseline at 4 specific time points (T2, T7, T9, and T13). The inferences at T2 and T13 compare the average initial and late (end line) change in the mean response relative to baseline for the intervention and control arms. The inferences at T7 and T9 are designed to investigate potential enhancements in the cross-arm differences in change relative to baseline at the 2 time points following reintroduction of the intervention. Secondary to these cross-arm comparisons, we also report Bonferroni-corrected (significance level .05/12) inferences to assess mean within-arm changes relative to baseline at each of the 12 subsequent time points (T2–T13). These analyses were conducted overall and separately for female and male participants.

All analyses were conducted as intent-to-treat and were completed using SAS (version 9.4; SAS Institute Inc).

### Ethical Considerations

The Institutional Review Board of Emory University (IRB00108404) and the Scientific and Ethics Review Unit of the Kenya Medical Research Institute (KEMRI; SERU: KEMRI/SERU/CGHR/11/3812) approved this study. Written informed consent was secured from one parent or guardian, and the participant provided their written assent prior to study baseline. To ensure participant privacy, datasets were deidentified; all participants were identified by a randomly assigned study identifier, which was the only identifier used in analysis datasets and to link data across time points and sources. Data were stored and transferred on encrypted devices. For each study visit, adolescent participants received KShs 500 (~US $5) as compensation for their time.

## Results

### Overview

**Figure 1:**
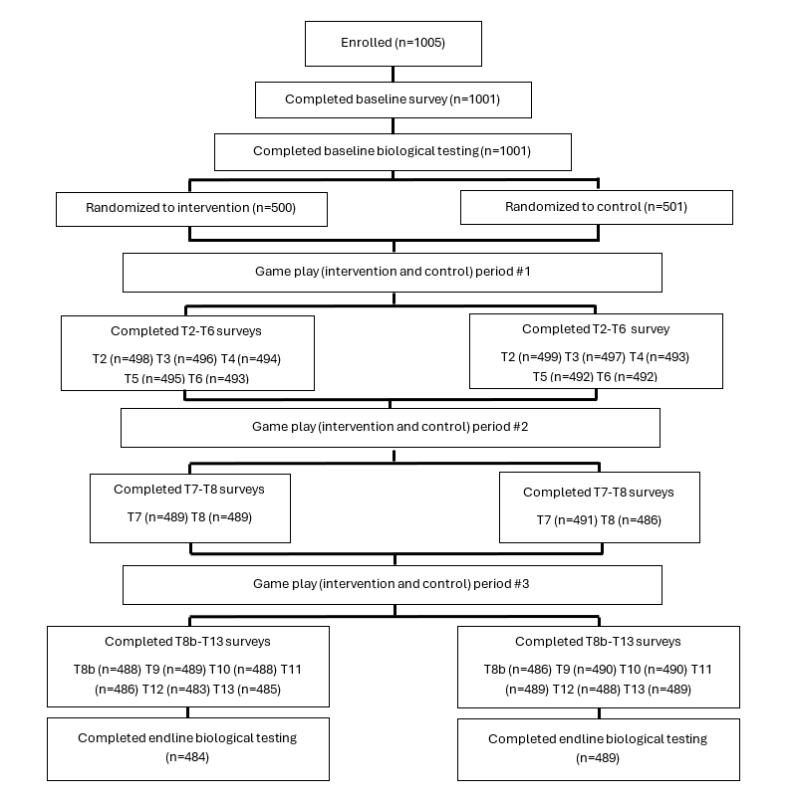
Trial flow diagram of study activities and participant totals for 45-month efficacy trial of a smartphone game (Tumaini) to increase age and condom use at first sex among young Kenyans.

A participant flowchart is shown in Figure 1, and baseline characteristics of the sample are presented in Table 2. Participants represented a range of socioeconomic and food security profiles. At end line, 97.8% of the sample (n=974) had been retained. There were no adverse events associated with study participation.

**Table 2:** Baseline characteristics of the intent-to-treat population (n=996) in the Tumaini efficacy trial.

Demographic characteristics			Intervention (n=499)		Control (n=497)
**Sex, n (%)**					
	Female	249 (50)		250 (50)	
	Male	250 (50)		247 (50)	
Age (years), mean (SD)			13.97 (0.57)		13.99 (0.54)
Enrolled in school, n (%)			499 (100)		493 (99)
**Socioeconomic status, n (%)**					
	Lower socioeconomic status	241 (49)		241 (49)	
	Higher socioeconomic status	254 (51)		251 (51)	
**Food insecurity, n (%)**					
	None	270 (54)		260 (52)	
	Low	138 (28)		149 (30)	
	Medium	73 (15)		73 (15)	
	High	18 (4)		15 (3)	
Living with HIV, n (%)			8 (2)		8 (2)
Experienced sexual debut, n (%)			76 (15)		76 (15)

Self-reported engagement in the intervention and control games was high at the end of each of the 3 intervention periods, and there was 100% exposure of each arm to their allocated game by T2. Participants in each arm dedicated comparable amounts of time to their assigned game across the 3 intervention periods, totaling 33.56 hours (SD 17.75) to Tumaini and 32.37 hours (SD 18.41) to the math game, indicating that the latter functioned as a highly effective attention-control condition. Fifty-eight percent of the control arm reported having played some of Tumaini by T9 (mean hours for those exposed: 19.77, SD 15.03) [19], with 35% exposed at T2 and 52% at T7.

We provide an overview of cross-arm differences in change since baseline at 4 time points (Table 3). Mean scores for each outcome at each time point and associated *P* values for within-arm change since baseline are presented in Tables S2-S9 in Multimedia Appendix 1.

**Table 3:** Cross-arm difference in mean change in proximal outcomes relative to baseline and associated *P* values at 4 time points in Tumaini efficacy trial.

Time point							T2^a^						T7^b^							T9^c^								T13^d^					
							Difference in change in mean score			*P* value			Difference in change in mean score				*P* value			Difference in change in mean score				*P* value			Difference in change in mean score					*P* value	
Mean age (years)							14.1 (0.56)			–^e^			15.6 (0.55)	–						16.4 (0.56)				–			17.7 (0.56)						–
**Behavioral intentions**																																	
	**Intention to use a condom at first sex**																																
				All		0.118			.005^f^			0.200				.008^f^			0.201				.01			0.164					.007^f^		
				Female		0.083			.02			0.088				.03			0.099				.02			0.103					.02		
				Male		0.055			.11			0.059				.11			0.045				.25			0.059					.14		
	**Intention to talk to a partner about HIV prevention**																																
				All		0.107			<.001^f^			0.085				<.001^f^			0.081				<.001^f^			0.076					<.001^f^		
				Female		0.115			<.001^f^			0.047				.11			0.048				.10			0.057					.05		
				Male		0.099			<.001^f^			0.123				<.001^f^			0.115				<.001^f^			0.095					.002^f^		
**Self-efficacy**																																	
	**Self-efficacy to use a condom correctly**																																
				All		0.215			<.001^f^			0.283				<.001^f^			0.273				<.001^f^			0.254					<.001^f^		
				Female		0.216			<.001^f^			0.278				<.001^f^			0.263				<.001^f^			0.283					<.001^f^		
				Male		0.213			<.001^f^			0.287				<.001^f^			0.282				<.001^f^			0.224					<.001^f^		
	**Self-efficacy to refuse unprotected sex**																																
				All		0.072			.006^f^			0.039				.13			0.042				.11			–0.037					.41		
				Female		0.107			.004^f^			0.028				.43			0.063				.09			0.040					.28		
				Male		0.037			.32			0.051				.17			0.021				.57			0.002					.96		
**Attitudes**																																	
	**Endorsement that condom use is a sign of respect**																																
				All		0.218			<.001^f^			0.201				<.001^f^			0.201				<.001^f^			0.164					<.001^f^		
				Female		0.234			<.001^f^			0.187				<.001^f^			0.201				<.001^f^			0.191					<.001^f^		
				Male		0.203			<.001^f^			0.214				<.001^f^			0.202				<.001^f^			0.138					<.001^f^		
	**Endorsement of gender parity in condom initiation**																																
				All		0.151			<.001^f^			0.205				<.001^f^			0.221				<.001^f^			0.192					<.001^f^		
				Female		0.146			<.001^f^			0.220				<.001^f^			0.218				<.001^f^			0.203					<.001^f^		
				Male		0.156			<.001^f^			0.189				<.001^f^			0.224				<.001^f^			0.181					<.001^f^		
**Knowledge**																																	
	**Knowledge of how to use a condom correctly**																																
			All		0.372			<.001^f^			0.429				<.001^f^			0.464				<.001^f^			0.458					<.001^f^			
			Female		0.376			<.001^f^			0.440				<.001^f^			0.462				<.001^f^			0.462					<.001^f^			
			Male		0.367			<.001^f^			0.417				<.001^f^			0.465				<.001^f^			0.453					<.001^f^			
	**Knowledge that condoms are effective for HIV prevention**																																
			All		0.096			.001^f^			0.093				.01^f^			0.085				.02			0.110					.003^f^			
			Female		0.116			.006^f^			0.123				.01			0.097				.05			0.158					.002^f^			
			Male		0.076			.07			0.059				.25			0.072				.17			0.062					.25			

^a^T2: second data collection time point.

^b^T7: 7^th^ data collection time point.

^c^T9: 9th data collection time point.

^d^T13: 13th data collection time point (end line).

^e^Not applicable.

^f^Indicates significance after Bonferroni correction (significance level .05/4, ie, .0125)

For each outcome, we present longitudinal plotlines below for all participants and stratified by gender. In the plots, solid lines track estimated mean responses (marked with triangles) for the intervention group over time; dashed lines (with circles) track estimated means for the control group. Solid markers (triangles for the intervention arm, circles for the control arm) indicate that the within-arm difference in mean response relative to baseline at that time point is significant after Bonferroni correction (ie, at α=.05/12 level); open symbols indicate lack of significance relative to baseline. Inferences addressing the 4 primary cross-arm comparisons of mean change relative to baseline are indicated with *P* values at the top of the plot. Asterisks after the *P* value indicate significance after Bonferroni correction (ie, at α=.05/4 level).

### Behavioral Intentions

#### Intention to Use a Condom at First Sex

**Figure 2:**
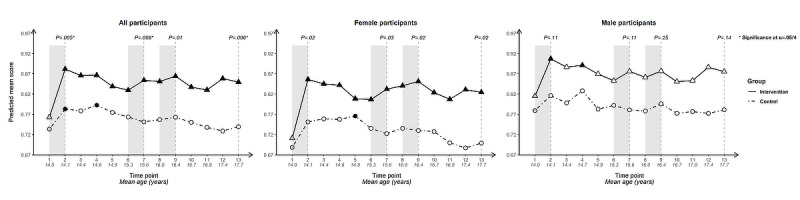
Longitudinal trajectory of intention to use a condom at first sex at 13 timepoints among all, female, and male participants not reporting sexual debut.

Figure 2 summarizes the results of the longitudinal analysis of the condom use at first sex behavioral intention outcome.

Cross-arm difference: when all participants were included in the analysis, there was significantly greater improvement in the intervention arm than the control arm immediately after initial intervention exposure (*P*=.005), following the second intervention period (ie, at T7; *P*=.008), and at end line (*P*=.007). Significance was marginal after Bonferroni correction following the third intervention period at T9 (*P*=.01) and not present when participants were stratified by gender, suggesting insufficient power once the sample was halved.

Within-arm change: solid triangles at T2-T13 for all participants indicate a Bonferroni-corrected statistically significant difference in the mean relative to baseline for the intervention arm at every subsequent time point. In addition to the steep uptick in mean score following initial intervention exposure, the intervention arm showed an uptick following the second and third intervention periods. In contrast, significant within-arm differences from baseline were present only occasionally in the control arm, with mean scores at end line closely resembling those at baseline.

Difference by gender: intention to use a condom at first sex was higher from baseline among male than female participants in both arms. Female intervention-arm participants saw a steeper uptick at T2 and significant improvement relative to baseline for all subsequent time points, such that it overtook the male control arm. After an initial upward trajectory, the control-arm plot trended downwards for female participants from T5 (age 14.8, SD 0.55).

#### Intention to Talk to a Partner About HIV Prevention

**Figure 3:**
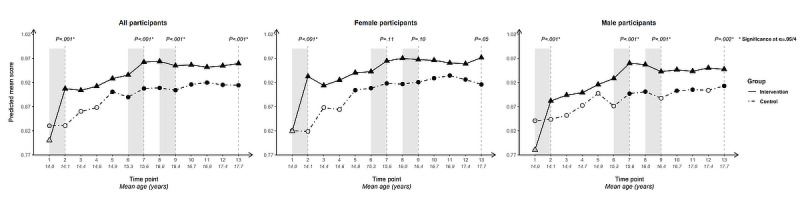
Longitudinal trajectory of intention to talk to a partner about HIV prevention at 13 time points among all, female, and male participants.

Cross-arm difference: significant cross-arm differences in increased intention to talk to a partner about HIV prevention (Figure 3) were present at all 4 time points for all participants and for male participants.

Despite an uptick in the intervention arm during the second intervention period, cross-arm differences were not significant at T7, T9, and end line for female participants. The plots suggest that this may be due to a combination of ceiling effects (mean scores in the intervention arm were extremely high from T7 [age 15.6, SD 0.55] onwards, limiting scope for improvement) and developmental gains in the control arm.

Within-arm change: for intervention-arm participants, within-arm change since baseline was significant for all time points for all female and male participants. For control-arm participants, difference relative to baseline mean became significant from T5 (age 14.9, SD 0.55) onwards for all and female participants. Male control-arm participants saw sporadic significant change since baseline from T7 (age 15.6, SD 0.56) onwards. Unlike intention to use a condom at first sex, mean scores followed an upward trajectory over time for both arms, suggesting a developmental effect.

A related outcome, self-efficacy to talk to a partner about HIV prevention (Figure S1 in Multimedia Appendix 2 and Table S10 in Multimedia Appendix 1), showed a similar trajectory to intention to talk to a partner about HIV prevention, with control-arm change since baseline for male participants achieving significance from mean age 14.9 (SD 0.56) years and end line values approximating those of the intervention arm.

### Self-Efficacy

#### Self-Efficacy to Use a Condom Correctly

**Figure 4:**
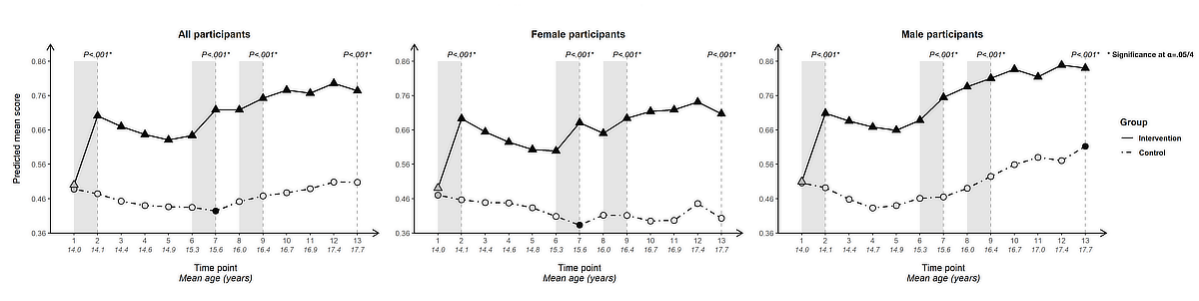
Longitudinal trajectory of self-efficacy to use a condom correctly at 13 time points among all, female, and male participants.

Cross-arm difference: baseline mean scores on self-efficacy to use a condom correctly (Figure 4) were lower and intervention-arm gains greater than for all other condom-related behavioral determinants except knowledge of correct condom use (which shows a similar trajectory, particularly in the intervention arm). Cross-arm differences in change since baseline were highly significant (*P*<.001) at all 4 time points for all participants and when stratified by gender.

Within-arm change from baseline was significant for intervention-arm participants at all subsequent time points, following a sharp uptick in mean score after the first intervention period and smaller upticks after the other intervention periods. For control-arm participants, a significant difference relative to baseline only occurred at one time point for each group: negatively at T7 (age 15.6, SD 0.55) for all and for female participants, and positively at end line for male participants.

Difference by gender: the overall upward trajectory among intervention-arm participants from T6 (age 15.4, SD 0.55) was steeper for male than for female participants. A similar upward trajectory was observed for male participants in the control arm, though at a much lower level, suggesting a developmental influence. The trajectory for female control-arm participants, by contrast, trended downwards.

#### Self-Efficacy to Refuse Unprotected Sex

**Figure 5:**
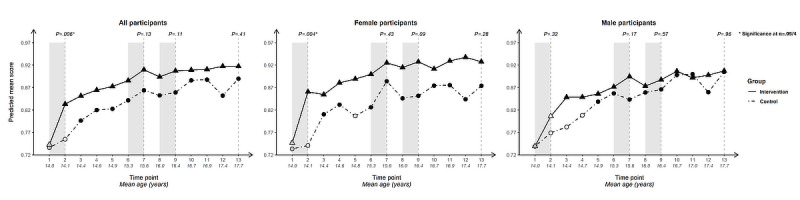
Longitudinal trajectory of self-efficacy to refuse unprotected sex at 13 time points among all, female, and male participants.

Cross-arm difference in increased self-efficacy to refuse unprotected sex (Figure 5) was significant following initial exposure to the intervention for all participants (*P*=.006) and for female participants (*P*=.004), but not for male participants (*P*=.32).

It was not significant at subsequent time points for any of the population groupings, despite upticks in the intervention arm following the second and third intervention periods. The upward trend in the intervention arm was mirrored by a similar trajectory, albeit at a generally lower level, in the control arm, suggesting a positive developmental influence for this outcome.

For the intervention arm, within-arm change since baseline was significant across all time points for all participants and when stratified by gender, except at T2 for male participants. The within-arm difference was significant for all control-arm participants from T3 (age 14.4, SD 0.56) onwards and for male participants from T5 onwards. For female participants, it was generally significant from T3 onwards.

Difference by gender: for male participants, the intervention-arm plotline was crossed by the control-arm plotline at T11 (age 17.0, SD 0.56; across all determinants, this was the only instance of this happening). End line values for both arms were nearly identical among male participants.

### Attitudes: Endorsement of “Using a Condom Is a Sign You Respect Your Partner” and “A Woman Can Suggest Using Condoms Just Like a Man Can”

**Figure 6:**
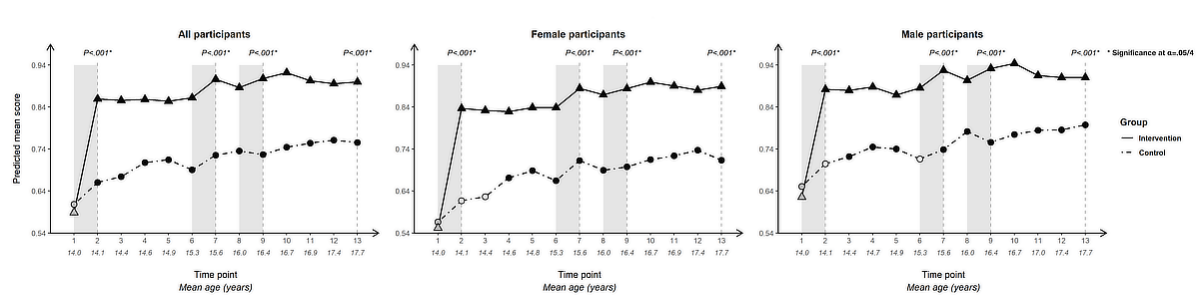
Longitudinal trajectory of endorsement of “Using a condom is a sign that you respect your partner” at 13 time points for all, female, and male participants.

**Figure 7:**
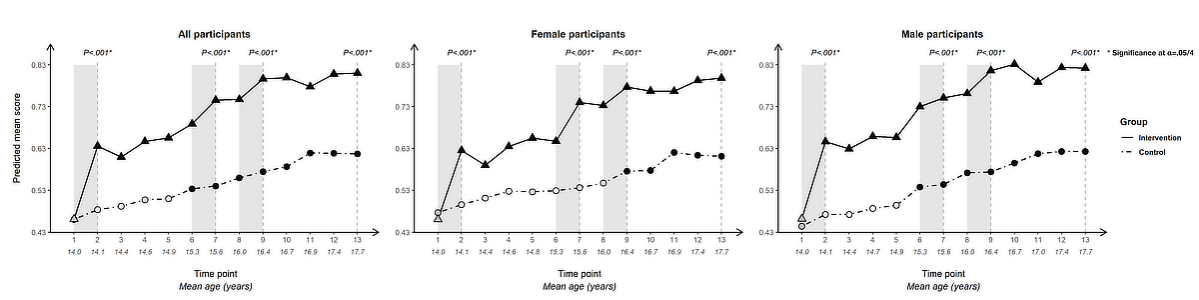
Longitudinal trajectory of endorsement of “A woman can suggest using condoms just like a man can” at 13 time points for all, female, and male participants.

The survey included 2 condom-related attitude items, asking participants to indicate their degree of agreement with “using a condom is a sign that you respect your partner” (Figure 6) and “a woman can suggest using a condom just like a man can” (Figure 7).

Cross-arm difference in change since baseline was highly significant for endorsement of both condom-related attitude questions at each time point and for all population groupings.

Significant within-arm change since baseline was present in the intervention arm at every time point for both questions and for all population groupings. The intervention-arm uptick following initial exposure to Tumaini was more pronounced for the first, gender-neutral question, with moderate increases across subsequent time points. For the second question, which challenged traditional cultural norms around women initiating condom use, the longitudinal trajectory for the intervention arm showed a steady rise from T2 that was reflected at lower mean scores but a similar slope in the control arm, suggesting a more pronounced intersection of intervention and developmental influences. Both questions saw an uptick in intervention-arm scores following the second and third intervention periods.

### Knowledge

#### Knowledge of How to Use a Condom Correctly

**Figure 8:**
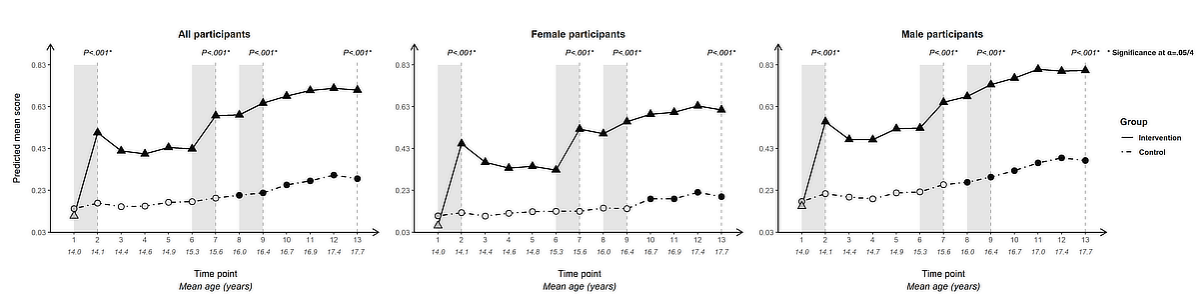
Longitudinal trajectory of knowledge of correct condom use at 13 time points for all, female, and male participants.

The 2 condom-related knowledge questions show variations in trajectory. For knowledge of correct condom use (Figure 8), the cross-arm change in score since baseline differed highly significantly (*P*<.001) at all 4 time points and across all population groupings.

Baseline scores were the lowest for any condom-use determinant, particularly among female participants, but saw a spike in the intervention arm following initial exposure that was built upon at the second and third intervention periods.

The within-arm difference since baseline in the intervention arm was significant at all 12 post-baseline time points across all population groupings. The control arm only saw significance from T8 (age 16.0, SD 0.55; T10: age 16.7, SD 0.55, for female participants) onward, with a slight upward trend at later ages, suggesting a mild developmental influence.

#### Knowledge That Condoms Are Effective at Preventing HIV

**Figure 9:**
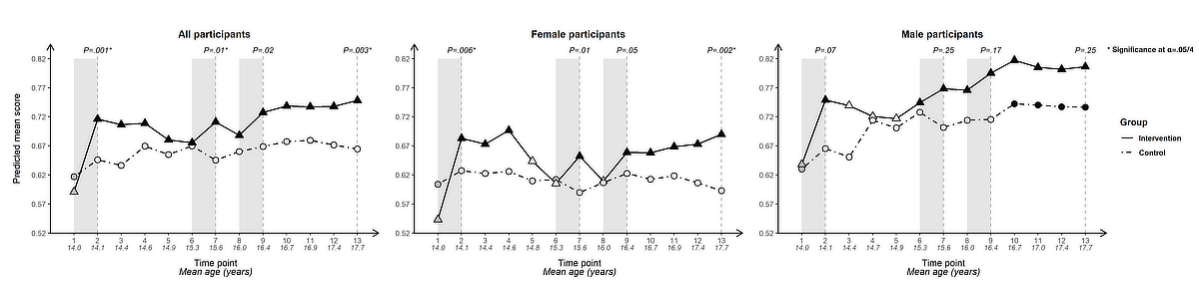
Longitudinal trajectory of knowledge that condoms are effective at preventing HIV at 13 time points for all, female, and male participants.

Cross-arm difference in change in knowledge that condoms are an effective way to prevent HIV (Figure 9) was significant following Bonferroni correction immediately after initial intervention exposure and at end line for all participants and for female participants, and at T7 for all participants. It was not significant at any time point for male participants, who had higher scores throughout than female participants.

The within-arm change since baseline was significant for the intervention arm at all time points for all participants.

This was true when stratifying by gender, except for at T5 and T6 for female participants and T3-T5 for male participants. In addition to a steep uptick after initial intervention exposure, the intervention arm saw upticks for the 2 subsequent intervention periods. This is particularly striking for female participants whose mean scores dip between intervention periods but recover following re-exposure to the intervention. Mean scores then stabilize with a slight upward trajectory from this point forward, while the control arm dips, indicating the value of repeated intervention exposure across mid-adolescence for female participants for this outcome. The control arm was nonsignificant for within-arm change since baseline at all time points except for male participants from T10 (age 16.7, SD 0.56) onward.

## Discussion

### Principal Findings

We evaluated the longitudinal impact of a theory-based choose-your-own-adventure smartphone game on proximal outcomes related to condom use across 45 months of mid-adolescence. These proximal outcomes are of particular importance given that assessment of primary behavioral outcomes, related to sexual debut, was not powered until end line, 45 months after participants’ initial exposure to Tumaini and 16 months after their most recent exposure. Intervention-arm participants devoted over 30 hours in total to playing Tumaini, while the control-arm game attracted similar levels of engagement, thereby functioning as an effective attention-control condition.

Cross-arm difference: despite cross-arm contamination, Tumaini significantly improved all intervention-targeted proximal influences of condom use relative to the control arm immediately after initial exposure. For almost all proximal outcomes, a significant cross-arm difference was also present at end line and, for most outcomes, at the 2 intervening time points when cross-arm comparison was undertaken, following re-exposure to the intervention.

Within-arm difference: for the intervention arm, when all participants were included in the analysis, the within-arm mean change since baseline was significant for every outcome at every time point. All outcomes saw a spike in intervention-arm mean score immediately after initial exposure to Tumaini. Repeat exposure to the intervention at ages 15.3 (SD 0.55) and 16.0 (SD 0.55) years reinforced these effects in almost every case.

These significant findings and their timing relative to the intervention periods provide compelling evidence of Tumaini’s impact and suggest that adolescents may benefit from even short-term exposure, though repeated exposure frequently sustained and strengthened effects. They are also consistent with our primary behavioral outcomes at end line; among participants reporting sexual debut between baseline and end line, 50% (41/82) of control-arm participants had unprotected first sex compared to 28% (23/81) of intervention-arm participants (relative risk [RR] 0.57, 95% CI 0.38-0.85) [19].

Multiple frameworks across health communication, social psychology, and behavioral science support a hierarchy of effects in behavioral interventions, for example [28], whereby knowledge is least resistant to change, followed by attitudes, self-efficacy, behavioral intentions, and finally behavior itself. In line with this hierarchy, while behavioral HIV prevention interventions for adolescents in sub-Saharan Africa have improved condom-related knowledge, attitudes, or self-efficacy, comparatively few have been shown in rigorous studies to reduce sexual risk by increasing condom use [5,29-31]. Our own findings are positive across the hierarchy of effects, from knowledge through attitudes, self-efficacy, and intentions to behavior.

Findings from the present analyses indicate that Tumaini’s condom-related content was most effective at strengthening self-efficacy to use a condom (especially among male participants), which is highly dependent on knowledge of how to use a condom correctly, and at informing positive condom-related attitudes. While findings are extremely positive across the board, they also highlight areas where the intervention could potentially be revised to achieve even stronger effects. For example, they suggest that Tumaini could do more to strengthen male participants’ self-efficacy to refuse unprotected sex. Trajectories for knowledge that condoms are an effective way to prevent HIV also differ substantially by gender. However, lower scores for female participants may relate less to condom efficacy, which is strongly emphasized throughout the intervention, than to the different levels of agency exercised by female versus male adolescents in condom use.

In addition to being the main strategy for HIV prevention for the population addressed in our trial, condoms are also the primary method of contraception [32]. Tumaini emphasizes the advantages of condoms for preventing pregnancy in addition to HIV. Pregnancy is an outcome that is a more immediate, and hence often more resonant, threat for adolescents—especially girls and young women—than HIV, even in high-prevalence parts of sub-Saharan Africa. Leveraging the long-term consequences of unplanned pregnancy for adolescents’ future plans and dreams in condom promotion efforts also has the advantage of being less likely to contribute to HIV stigma. It is notable that trajectories for pregnancy-related proximal outcomes (Figures S2-S4 in Multimedia Appendix 2) were very similar to those for HIV prevention.

In their 2010 systematic review and meta-analysis of HIV prevention for sub-Saharan African youth, Michielsen et al [33] found that effects on condom use were larger in male participants in almost all studies where these were reported as significant. Gender stratification of our primary behavioral outcomes at end line, in contrast, revealed a stronger impact on female participants, for whom the risk of unprotected first sex was reduced significantly from 57% (21/37) in the control arm to 21% (7/33) in the intervention arm (RR 0.37, 95% CI 0.18-0.76) [19]. Male participants also saw a reduction, but it was nonsignificant, from 44% (20/45) in the control arm to 33% (16/48) in the intervention arm (RR 0.75, 95% CI 0.45-1.26) despite them scoring higher in the present analyses at every time point for intention to use a condom at first sex. Intention may be lower for many adolescent girls and young women in Kenya because they envisage their sexual debut happening within marriage, a context in which they foresee less need to rely on a condom. Lower intervention impact on male participants’ condom use at first sex suggests that the translation of intention into behavior may operate differently for male than for female adolescents. It is possible that lower self-efficacy to refuse unprotected sex may be a factor and/or that male participants struggle in the emotionally charged context of first sex when performance anxiety may be exacerbated by condom use.

Alongside those referenced above, multiple structural and contextual factors have been identified as potentially militating against translation of condom-use proximal outcomes up the hierarchy of effects, from knowledge to behavior. Sociocultural factors include, for example, the association of condoms with immorality; gender norms promoting “submissiveness” in adolescent girls and young women; perceptions among young men that condoms reduce sexual pleasure; and a range of myths and misconceptions [15,16,34,35]. Among behavioral theories, social cognitive theory, in which Tumaini is grounded, has the advantage of foregrounding the role played by the social environment in individual behavior. Tumaini’s narrative-based approach, drawing on research on thousands of stories contributed by young Africans to scriptwriting competitions [15,16,36], ensures that contextual and structural factors are extensively engaged with.

In addition, although delivered at the individual level, Tumaini either supports or has the potential to support engagement at higher socioecological levels of analysis. It catalyzes considerable household (including parent-child) interaction [37], which is modeled and nurtured in the game by the presence of parent, other adults, and older peer characters who function as “virtual mentors.” Further, the high levels of engagement it causes and sustains suggest that, when widely disseminated, its effects could extend to the community level with the potential to support normative change. Depending on population needs, moreover, its effects could be enhanced through dissemination in combination with structural or biomedical programming, alongside anticipated direct-to-consumer dissemination via an app store.

By 2030, smartphones will account for over 80% of all mobile connections in sub-Saharan Africa [7]. Although growth is uneven and there are cross-national, urban-rural, and gender disparities in mobile phone ownership generally and particularly for more expensive phones, penetration is especially rapid among young people [38,39]. At present, over 3 quarters of all mobile phones in Kenya are smartphones (compared to 61% across all of sub-Saharan Africa). While there is no gender disparity in ownership of mobile phones in general in Kenya, there is one for smartphones (49% of men compared to 43% of women own one). In rural areas, a combination of lower data access and cost limits smartphone ownership. Nonetheless, smartphone access and equity in access are increasing in Kenya and across sub-Saharan Africa, and this trajectory is unlikely to change in the near future.

With access barriers in mind, we built Tumaini for a low-cost smartphone as an app that does not need ongoing data access or regular updates. It is designed to be played by adolescents on the phones of parents, siblings, and other family members, or on their own phones or those of their friends; hence, players need access to a smartphone, but they do not need to own one themselves. At the study end line in 2024, 11% of efficacy trial participants (n=106) indicated that no one in their household owned a smartphone, and 17% (n=168) reported owning their own smartphone (though there was a gender disparity, with 21% of boys compared to 14% of girls owning one). Tumaini’s scalability and impact outside of efficacy trial conditions warrants further investigation (and ongoing investigation as smartphone access increases).

### Strengths and Limitations

Some longitudinal observational studies have followed cohorts of young Africans through adolescence, tracking sexual and reproductive health outcomes [40,41]. However, longitudinal intervention evaluations are rare. An exception is “Let Us Protect Our Future!” with follow-ups at 3, 6, 12, 42, and 54 months [42,43]. Our efficacy trial is notable for the length and frequency of follow-up (13 surveys over 45 months), in addition to its extremely high levels of participant retention (97.5% at end line), its effective attention-control condition, and its high and sustained intervention engagement.

The longitudinal trajectories presented here allow us to observe Tumaini’s influence on a range of theory-based, condom-related proximal outcomes across mid-adolescence and across the 3 intervention periods. The within-arm comparisons, with plotlines incorporating intervention periods, provide invaluable contextualization for the cross-arm comparative data and facilitate identification of gender-specific and developmental influences. These analyses thus provide richer insights into the effects of Tumaini, including how these may be optimized, than end line estimates of relative risk alone.

In addition to these strengths, the study has limitations. First, there is a risk that repeated participant exposure to the same survey items over time might blunt the sensitivity of those questions. However, cross-arm differences, upticks in intervention-arm mean scores at each intervention period, and diversity in longitudinal trajectory by outcome suggest that this was not a major concern in this study. Second, while we drew our survey items from scales that had been used and, where possible, also validated with similar populations, we were unable to use the complete validated scales due to the age of our participants (some as young as 12 at baseline) and the risk of overburdening them with an extensive questionnaire or exposing them to non–age-appropriate content. Third, by their nature, our proximal outcomes are self-reported. Specifically, self-efficacy to use a condom correctly and knowledge of how to use a condom correctly are self-assessments, and it is possible that these measures may not ultimately mediate correct condom use in real-life circumstances. Fourth, the study provided participants with smartphones with parental restrictions blocking internet access; additional research is needed to assess Tumaini’s effectiveness under more “real-world” conditions. We are planning a hybrid type 2 effectiveness-implementation study. Fifth, as parents self-selected to learn more about the study, the sample may be biased toward participants whose parents are more open to sexual and reproductive health education for adolescents. Finally, cross-arm contamination may have reduced our ability to detect some intervention effects.

### Future Research

Ongoing qualitative research with this cohort may shed additional light on contextual factors informing these trajectories. Mediation analysis currently underway will allow us to pinpoint which proximal outcomes mediated our behavioral outcomes at end line for those who had reached sexual debut; there is no scope for mediation analysis at earlier time points because these outcomes are developmentally influenced, resulting in small cell sizes at younger participant ages. Through this additional research, we will seek to optimize Tumaini prior to our planned hybrid type 2 effectiveness-implementation trial.

### Conclusions

Tumaini significantly improved theory-based proximal influences of condom use, with effects sustained 45 months post-initial exposure and 16 months post-most recent exposure. Spikes in intervention-arm scores immediately after initial exposure indicate that adolescents benefit from even short-term exposure, though repeated exposure frequently sustained and reinforced effects. At a time when threats to global HIV funding highlight the necessity of preventing new infections among young people, study findings support the promise of reaching adolescents at an early age with information and skills to support a safer sexual debut. In the context of rapidly increasing access to smartphones in sub-Saharan Africa and at a time when cost-effectiveness is at a premium, Tumaini has potential for high scalability and impact on condom-related outcomes.

## Appendix

Multimedia Appendix 1 Supplemental tables with additional outcomes and mean outcome scores at 13 time points and associated within- and cross-arm change.

Multimedia Appendix 2 Longitudinal trajectories of additional survey items with relevance to condom use at 13 time points among all, female, and male participants.

Multimedia Appendix 3 CONSORT-eHEALTH checklist (V 1.6.1).

Multimedia Appendix 4 Higher-resolution versions of figures 2-9.

## Abbreviations

KEMRI: Kenya Medical Research Institute
RR: relative risk

## Data Availability

Data underlying these analyses are not publicly available as the analyses are ongoing. Data are available from the corresponding author on reasonable request.
