# Supplementary material for: Longitudinal Effects of a Smartphone Game (Tumaini) for HIV Prevention Among Kenyan Adolescents: 45-Month Trajectories of Condom Use–Related Proximal Outcomes From a Randomized Controlled Trial
Source: J Med Internet Res. 2026 Mar 10;28:e83982. doi: 10.2196/83982 (PMC13014075; doi:10.2196/83982)
Supplement: Multimedia Appendix 1 [file jmir_v28i1e83982_app1.docx]

**Table S1:** Additional condom-related proximal outcomes, question wording, and response options

| **Proximal outcome** | **Condom-related proximal outcome** | **Question wording** | **Response options** |
| --- | --- | --- | --- |
| Behavioral intention | Intention to talk to a partner about pregnancy prevention | “*Imagine that either in the future or now you are thinking about having sex with someone and you are not ready to become a parent*.  Would you talk with him/her about preventing pregnancy?” | Definitely yes, maybe yes, maybe no, definitely no |
| Self-efficacy | Self-efficacy to talk to a partner about HIV prevention | *Imagine that either in the future or now you are thinking about having sex with someone and you are not ready to become a parent*  “If I wanted to talk to him/her about preventing pregnancy, I am sure that I could do that.”  How much do you agree with this statement? | I strongly agree, I agree, I disagree, I strongly agree |
|  | Self-efficacy to discuss pregnancy prevention with a partner | *Imagine that either in the future or now you are thinking about having sex with someone and you are not ready to become a parent*  “If I wanted to talk to him/her about preventing pregnancy, I am sure that I could do that.”  How much do you agree with this statement? | I strongly agree, I agree, I disagree, I strongly agree |
| Knowledge | Knowledge that condoms are an effective way to prevent pregnancy | "Are condoms an effective way to prevent pregnancy?” | Yes, no, I don’t know |

**Table S2.** Mean scores in intention to use a condom at first sex and *P*-values for difference from baseline mean score by study arm for all participants.

|  | **All participants** | | | | **Female participants** | | | | **Male participants** | | | |
| --- | --- | --- | --- | --- | --- | --- | --- | --- | --- | --- | --- | --- |
|  | **Intervention** | | **Control** | | **Intervention** | | **Control** | | **Intervention** | | **Control** | |
|  | Mean score | *P-*value | Mean score | *P-*value | Mean score | *P-*value | Mean score | *P-*value | Mean score | *P-*value | Mean score | *P-*value |
| **T1** | 0.763 |  | 0.733 |  | 0.711 |  | 0.688 |  | 0.815 |  | 0.779 |  |
| **T2** | 0.882 | *<.0001** | 0.783 | *0.0032** | 0.856 | *<.0001** | 0.751 | *0.0094* | 0.907 | *0.0002** | 0.816 | *0.1198* |
| **T3** | 0.866 | *<.0001** | 0.779 | *0.0079* | 0.845 | *<.0001** | 0.759 | *0.0056* | 0.887 | *0.0047* | 0.798 | *0.3937* |
| **T4** | 0.867 | *<.0001** | 0.793 | *0.0007** | 0.842 | *<.0001** | 0.758 | *0.0059* | 0.891 | *0.0032** | 0.828 | *0.0405* |
| **T5** | 0.839 | *0.0003** | 0.775 | *0.0273* | 0.809 | *0.0013** | 0.766 | *0.0036** | 0.869 | *0.0565* | 0.783 | *0.8875* |
| **T6** | 0.830 | *0.0011** | 0.764 | *0.1064* | 0.807 | *0.0012** | 0.736 | *0.0957* | 0.853 | *0.1791* | 0.792 | *0.5968* |
| **T7** | 0.854 | *<.0001** | 0.751 | *0.357* | 0.832 | *<.0001** | 0.722 | *0.2254* | 0.876 | *0.0182* | 0.781 | *0.9545* |
| **T8** | 0.851 | *<.0001** | 0.757 | *0.2369* | 0.840 | *<.0001** | 0.735 | *0.0986* | 0.861 | *0.09* | 0.778 | *0.9718* |
| **T9** | 0.864 | *<.0001** | 0.763 | *0.166* | 0.851 | *<.0001** | 0.730 | *0.1715* | 0.877 | *0.0197* | 0.796 | *0.5686* |
| **T10** | 0.837 | *0.0003** | 0.750 | *0.408* | 0.824 | *<.0001** | 0.728 | *0.1752* | 0.851 | *0.2415* | 0.772 | *0.8084* |
| **T11** | 0.830 | *0.001** | 0.738 | *0.8331* | 0.807 | *0.0008** | 0.700 | *0.704* | 0.853 | *0.1874* | 0.777 | *0.9352* |
| **T12** | 0.858 | *<.0001** | 0.729 | *0.8363* | 0.831 | *<.0001** | 0.687 | *0.9636* | 0.886 | *0.0075* | 0.772 | *0.8149* |
| **T13** | 0.850 | *<.0001** | 0.739 | *0.7833* | 0.824 | *<.0001** | 0.699 | *0.7478* | 0.876 | *0.0288* | 0.781 | *0.9485* |

* indicates within-arm significance after Bonferroni correction (α=.05/12)

**Table S3.** Mean scores in intention to talk to a partner about HIV prevention and *P*-values for difference from baseline mean score by study arm for all participants.

|  | **All participants** | | | | **Female participants** | | | | **Male participants** | | | |
| --- | --- | --- | --- | --- | --- | --- | --- | --- | --- | --- | --- | --- |
|  | **Intervention** | | **Control** | | **Intervention** | | **Control** | | **Intervention** | | **Control** | |
|  | Mean score | *P-value* | Mean score | *P-value* | Mean score | *P-value* | Mean score | *P-value* | Mean score | *P-value* | Mean score | *P-*value |
| **T1** | 0.800 |  | 0.830 |  | 0.819 |  | 0.820 |  | 0.780 |  | 0.841 |  |
| **T2** | 0.907 | *<.0001** | 0.830 | *0.9858* | 0.932 | *<.0001** | 0.818 | *0.9305* | 0.882 | *<.0001** | 0.843 | *0.9002* |
| **T3** | 0.904 | *<.0001** | 0.860 | *0.0352* | 0.914 | *<.0001** | 0.868 | *0.0186* | 0.894 | *<.0001** | 0.852 | *0.5709* |
| **T4** | 0.912 | *<.0001** | 0.868 | *0.0099* | 0.925 | *<.0001** | 0.864 | *0.0469* | 0.899 | *<.0001** | 0.872 | *0.1036* |
| **T5** | 0.928 | *<.0001** | 0.901 | *<.0001** | 0.940 | *<.0001** | 0.904 | *<.0001** | 0.916 | *<.0001** | 0.897 | *0.0066* |
| **T6** | 0.935 | *<.0001** | 0.889 | *<.0001** | 0.942 | *<.0001** | 0.908 | *<.0001** | 0.928 | *<.0001** | 0.871 | *0.1538* |
| **T7** | 0.962 | *<.0001** | 0.907 | *<.0001** | 0.964 | *<.0001** | 0.918 | *<.0001** | 0.960 | *<.0001** | 0.897 | *0.003** |
| **T8** | 0.963 | *<.0001** | 0.909 | *<.0001** | 0.970 | *<.0001** | 0.917 | *<.0001** | 0.957 | *<.0001** | 0.901 | *0.0024** |
| **T9** | 0.955 | *<.0001** | 0.904 | *<.0001** | 0.967 | *<.0001** | 0.920 | *<.0001** | 0.942 | *<.0001** | 0.887 | *0.036** |
| **T10** | 0.956 | *<.0001** | 0.916 | *<.0001** | 0.966 | *<.0001** | 0.929 | *<.0001** | 0.946 | *<.0001** | 0.903 | *0.0026** |
| **T11** | 0.952 | *<.0001** | 0.920 | *<.0001** | 0.960 | *<.0001** | 0.934 | *<.0001** | 0.943 | *<.0001** | 0.905 | *0.0035** |
| **T12** | 0.954 | *<.0001** | 0.915 | *<.0001** | 0.959 | *<.0001** | 0.926 | *<.0001** | 0.950 | *<.0001** | 0.904 | *0.0042** |
| **T13** | 0.959 | *<.0001** | 0.914 | *<.0001** | 0.971 | *<.0001** | 0.916 | *<.0001** | 0.947 | *<.0001** | 0.913 | *0.0004** |

* indicates within-arm significance after Bonferroni correction (α=.05/12)

**Table S4** Mean scores in self-efficacy to use a condom correctly and *P*-values for difference from baseline mean score by study arm for all participants.

|  | **All participants** | | | | **Female participants** | | | | **Male participants** | | | |
| --- | --- | --- | --- | --- | --- | --- | --- | --- | --- | --- | --- | --- |
|  | **Intervention** | | **Control** | | **Intervention** | | **Control** | | **Intervention** | | **Control** | |
|  | Mean score | *P-value* | Mean score | *P-value* | Mean score | *P-value* | Mean score | *P-value* | Mean score | *P-value* | Mean score | *P-*value |
| **T1** | 0.500 |  | 0.488 |  | 0.491 |  | 0.470 |  | 0.510 |  | 0.506 |  |
| **T2** | 0.701 | *<.0001** | 0.474 | *0.4307* | 0.693 | *<.0001** | 0.457 | *0.5648* | 0.709 | *<.0001** | 0.492 | *0.5814* |
| **T3** | 0.670 | *<.0001** | 0.453 | *0.0549* | 0.654 | *<.0001** | 0.449 | *0.3898* | 0.686 | *<.0001** | 0.458 | *0.0691* |
| **T4** | 0.647 | *<.0001** | 0.440 | *0.0093* | 0.625 | *<.0001** | 0.447 | *0.3885* | 0.668 | *<.0001** | 0.433 | *0.0043* |
| **T5** | 0.631 | *<.0001** | 0.436 | *0.0056* | 0.603 | *0.0001** | 0.434 | *0.1733* | 0.659 | *<.0001** | 0.440 | *0.0101* |
| **T6** | 0.644 | *<.0001** | 0.435 | *0.0074* | 0.599 | *0.0003** | 0.408 | *0.0256* | 0.688 | *<.0001** | 0.461 | *0.1161* |
| **T7** | 0.718 | *<.0001** | 0.424 | *0.0013** | 0.681 | *<.0001** | 0.383 | *0.003** | 0.755 | *<.0001** | 0.465 | *0.1224* |
| **T8** | 0.718 | *<.0001** | 0.451 | *0.0557* | 0.650 | *<.0001** | 0.412 | *0.0386* | 0.786 | *<.0001** | 0.490 | *0.5483* |
| **T9** | 0.753 | *<.0001** | 0.468 | *0.3233* | 0.694 | *<.0001** | 0.411 | *0.036* | 0.810 | *<.0001** | 0.525 | *0.4972* |
| **T10** | 0.776 | *<.0001** | 0.477 | *0.5985* | 0.714 | *<.0001** | 0.395 | *0.0088* | 0.836 | *<.0001** | 0.559 | *0.0489* |
| **T11** | 0.767 | *<.0001** | 0.489 | *0.9602* | 0.719 | *<.0001** | 0.397 | *0.0088* | 0.815 | *<.0001** | 0.580 | *0.0096* |
| **T12** | 0.795 | *<.0001** | 0.508 | *0.3235* | 0.741 | *<.0001** | 0.446 | *0.4006* | 0.849 | *<.0001** | 0.571 | *0.0284* |
| **T13** | 0.774 | *<.0001** | 0.508 | *0.3413* | 0.707 | *<.0001** | 0.403 | *0.025* | 0.840 | *<.0001** | 0.612 | *0.0002** |

* indicates within-arm significance after Bonferroni correction (α=.05/12)

**Table S5.** Mean scores in self-efficacy to refuse unprotected sex and *P*-values for difference from baseline mean score by study arm for all participants.

|  | **All participants** | | | | **Female participants** | | | | **Male participants** | | | |
| --- | --- | --- | --- | --- | --- | --- | --- | --- | --- | --- | --- | --- |
|  | **Intervention** | | **Control** | | **Intervention** | | **Control** | | **Intervention** | | **Control** | |
|  | Mean score | *P-value* | Mean score | *P-value* | Mean score | *P-value* | Mean score | *P-value* | Mean score | *P-value* | Mean score | *P-*value |
| **T1** | 0.743 |  | 0.736 |  | 0.746 |  | 0.733 |  | 0.739 |  | 0.739 |  |
| **T2** | 0.833 | *<.0001** | 0.755 | *0.3236* | 0.861 | *<.0001** | 0.741 | *0.7884* | 0.806 | *0.0141* | 0.769 | *0.2258* |
| **T3** | 0.851 | *<.0001** | 0.796 | *0.0007** | 0.854 | *<.0001** | 0.810 | *0.0028** | 0.849 | *<.0001** | 0.782 | *0.0769* |
| **T4** | 0.865 | *<.0001** | 0.820 | *<.0001** | 0.880 | *<.0001** | 0.832 | *<.0001** | 0.849 | *<.0001** | 0.808 | *0.0048* |
| **T5** | 0.872 | *<.0001** | 0.823 | *<.0001** | 0.889 | *<.0001** | 0.807 | *0.0069* | 0.856 | *<.0001** | 0.839 | *<.0001** |
| **T6** | 0.886 | *<.0001** | 0.842 | *<.0001** | 0.900 | *<.0001** | 0.826 | *0.0008** | 0.872 | *<.0001** | 0.857 | *<.0001** |
| **T7** | 0.910 | *<.0001** | 0.864 | *<.0001** | 0.925 | *<.0001** | 0.884 | *<.0001** | 0.895 | *<.0001** | 0.843 | *<.0001** |
| **T8** | 0.894 | *<.0001** | 0.853 | *<.0001** | 0.915 | *<.0001** | 0.846 | *<.0001** | 0.874 | *<.0001** | 0.859 | *<.0001** |
| **T9** | 0.907 | *<.0001** | 0.859 | *<.0001** | 0.928 | *<.0001** | 0.852 | *<.0001** | 0.887 | *<.0001** | 0.866 | *<.0001** |
| **T10** | 0.909 | *<.0001** | 0.886 | *<.0001** | 0.912 | *<.0001** | 0.874 | *<.0001** | 0.906 | *<.0001** | 0.898 | *<.0001** |
| **T11** | 0.911 | *<.0001** | 0.888 | *<.0001** | 0.929 | *<.0001** | 0.875 | *<.0001** | 0.892 | *<.0001** | 0.900 | *<.0001** |
| **T12** | 0.918 | *<.0001** | 0.852 | *<.0001** | 0.938 | *<.0001** | 0.844 | *<.0001** | 0.898 | *<.0001** | 0.860 | *<.0001** |
| **T13** | 0.917 | *<.0001** | 0.890 | *<.0001** | 0.928 | *<.0001** | 0.874 | *<.0001** | 0.907 | *<.0001** | 0.905 | *<.0001** |

* indicates within-arm significance after Bonferroni correction (α=.05/12)

**Table S6.** Mean scores in endorsement of “*using a condom is a sign that you respect your partner*” and *P*-values for difference from baseline mean score by study arm for all participants.

|  | **All participants** | | | | **Female participants** | | | | **Male participants** | | | |
| --- | --- | --- | --- | --- | --- | --- | --- | --- | --- | --- | --- | --- |
|  | **Intervention** | | **Control** | | **Intervention** | | **Control** | | **Intervention** | | **Control** | |
|  | Mean score | *P-value* | Mean score | *P-value* | Mean score | *P-value* | Mean score | *P-value* | Mean score | *P-value* | Mean score | *P-*value |
| **T1** | 0.589 |  | 0.608 |  | 0.552 |  | 0.566 |  | 0.625 |  | 0.651 |  |
| **T2** | 0.859 | *<.0001** | 0.661 | *0.0011** | 0.837 | *<.0001** | 0.617 | *0.0419* | 0.882 | *<.0001** | 0.705 | *0.0073* |
| **T3** | 0.856 | *<.0001** | 0.674 | *<.0001** | 0.832 | *<.0001** | 0.626 | *0.013* | 0.880 | *<.0001** | 0.722 | *0.0006** |
| **T4** | 0.858 | *<.0001** | 0.708 | *<.0001** | 0.829 | *<.0001** | 0.671 | *<.0001** | 0.888 | *<.0001** | 0.745 | *<.0001** |
| **T5** | 0.854 | *<.0001** | 0.714 | *<.0001** | 0.839 | *<.0001** | 0.688 | *<.0001** | 0.869 | *<.0001** | 0.740 | *0.0001** |
| **T6** | 0.862 | *<.0001** | 0.690 | *<.0001** | 0.839 | *<.0001** | 0.665 | *<.0001** | 0.885 | *<.0001** | 0.716 | *0.0049* |
| **T7** | 0.906 | *<.0001** | 0.725 | *<.0001** | 0.885 | *<.0001** | 0.712 | *<.0001** | 0.927 | *<.0001** | 0.739 | *0.0002** |
| **T8** | 0.886 | *<.0001** | 0.736 | *<.0001** | 0.869 | *<.0001** | 0.690 | *<.0001** | 0.903 | *<.0001** | 0.782 | *<.0001** |
| **T9** | 0.908 | *<.0001** | 0.727 | *<.0001** | 0.884 | *<.0001** | 0.697 | *<.0001** | 0.932 | *<.0001** | 0.756 | *<.0001** |
| **T10** | 0.922 | *<.0001** | 0.745 | *<.0001** | 0.900 | *<.0001** | 0.715 | *<.0001** | 0.944 | *<.0001** | 0.775 | *<.0001** |
| **T11** | 0.903 | *<.0001** | 0.754 | *<.0001** | 0.890 | *<.0001** | 0.724 | *<.0001** | 0.915 | *<.0001** | 0.784 | *<.0001** |
| **T12** | 0.895 | *<.0001** | 0.761 | *<.0001** | 0.880 | *<.0001** | 0.738 | *<.0001** | 0.911 | *<.0001** | 0.785 | *<.0001** |
| **T13** | 0.900 | *<.0001** | 0.755 | *<.0001** | 0.889 | *<.0001** | 0.713 | *<.0001** | 0.911 | *<.0001** | 0.797 | *<.0001** |

* indicates within-arm significance after Bonferroni correction (α=.05/12)

**Table S7.** Mean scores in endorsement of “*A woman can suggest using condoms just like a man can*” and *P*-values for difference from baseline mean score by study arm for all participants.

|  | **All participants** | | | | **Female participants** | | | | **Male participants** | | | |
| --- | --- | --- | --- | --- | --- | --- | --- | --- | --- | --- | --- | --- |
|  | **Intervention** | | **Control** | | **Intervention** | | **Control** | | **Intervention** | | **Control** | |
|  | Mean score | *P-value* | Mean score | *P-value* | Mean score | *P-value* | Mean score | *P-value* | Mean score | *P-value* | Mean score | *P-*value |
| **T1** | 0.461 |  | 0.460 |  | 0.460 |  | 0.477 |  | 0.462 |  | 0.444 |  |
| **T2** | 0.636 | *<.0001** | 0.484 | *0.1699* | 0.625 | *<.0001** | 0.496 | *0.4514* | 0.646 | *<.0001** | 0.472 | *0.2214* |
| **T3** | 0.610 | *<.0001** | 0.492 | *0.0696* | 0.590 | *<.0001** | 0.512 | *0.1534* | 0.629 | *<.0001** | 0.472 | *0.2478* |
| **T4** | 0.647 | *<.0001** | 0.507 | *0.0113* | 0.635 | *<.0001** | 0.528 | *0.0575* | 0.659 | *<.0001** | 0.487 | *0.0893* |
| **T5** | 0.656 | *<.0001** | 0.510 | *0.0067* | 0.655 | *<.0001** | 0.526 | *0.0644* | 0.656 | *<.0001** | 0.494 | *0.0444* |
| **T6** | 0.689 | *<.0001** | 0.534 | *0.0001** | 0.647 | *<.0001** | 0.529 | *0.0459* | 0.730 | *<.0001** | 0.538 | *0.0005** |
| **T7** | 0.746 | *<.0001** | 0.540 | *<.0001** | 0.740 | *<.0001** | 0.536 | *0.0272* | 0.751 | *<.0001** | 0.544 | *0.0002** |
| **T8** | 0.747 | *<.0001** | 0.560 | *<.0001** | 0.733 | *<.0001** | 0.548 | *0.0081* | 0.761 | *<.0001** | 0.572 | *<.0001** |
| **T9** | 0.797 | *<.0001** | 0.575 | *<.0001** | 0.777 | *<.0001** | 0.575 | *0.0003** | 0.816 | *<.0001** | 0.574 | *<.0001** |
| **T10** | 0.800 | *<.0001** | 0.586 | *<.0001** | 0.768 | *<.0001** | 0.578 | *0.0006** | 0.831 | *<.0001** | 0.595 | *<.0001** |
| **T11** | 0.778 | *<.0001** | 0.619 | *<.0001** | 0.767 | *<.0001** | 0.621 | *<.0001** | 0.789 | *<.0001** | 0.618 | *<.0001** |
| **T12** | 0.808 | *<.0001** | 0.619 | *<.0001** | 0.793 | *<.0001** | 0.614 | *<.0001** | 0.824 | *<.0001** | 0.623 | *<.0001** |
| **T13** | 0.810 | *<.0001** | 0.617 | *<.0001** | 0.798 | *<.0001** | 0.612 | *<.0001** | 0.822 | *<.0001** | 0.623 | *<.0001** |

* indicates within-arm significance after Bonferroni correction (α=.05/12)

**Table S8.** Mean scores in knowledge of correct condom use and *P*-values for difference from baseline mean score by study arm for all participants.

|  | **All participants** | | | | **Female participants** | | | | **Male participants** | | | |
| --- | --- | --- | --- | --- | --- | --- | --- | --- | --- | --- | --- | --- |
|  | **Intervention** | | **Control** | | **Intervention** | | **Control** | | **Intervention** | | **Control** | |
|  | Mean score | *P-value* | Mean score | *P-value* | Mean score | *P-value* | Mean score | *P-value* | Mean score | *P-value* | Mean score | *P-*value |
| **T1** | 0.108 |  | 0.142 |  | 0.061 |  | 0.108 |  | 0.154 |  | 0.177 |  |
| **T2** | 0.506 | *<.0001** | 0.169 | *0.0773* | 0.454 | *<.0001** | 0.124 | *0.3731* | 0.558 | *<.0001** | 0.214 | *0.1239* |
| **T3** | 0.419 | *<.0001** | 0.152 | *0.5075* | 0.364 | *<.0001** | 0.107 | *0.9775* | 0.473 | *<.0001** | 0.198 | *0.3772* |
| **T4** | 0.405 | *<.0001** | 0.155 | *0.4682* | 0.337 | *<.0001** | 0.120 | *0.5827* | 0.472 | *<.0001** | 0.189 | *0.6261* |
| **T5** | 0.435 | *<.0001** | 0.173 | *0.0779* | 0.345 | *<.0001** | 0.128 | *0.3481* | 0.525 | *<.0001** | 0.218 | *0.1289* |
| **T6** | 0.428 | *<.0001** | 0.176 | *0.059* | 0.328 | *<.0001** | 0.130 | *0.3174* | 0.528 | *<.0001** | 0.223 | *0.1042* |
| **T7** | 0.588 | *<.0001** | 0.193 | *0.0071* | 0.523 | *<.0001** | 0.130 | *0.3478* | 0.651 | *<.0001** | 0.257 | *0.0069* |
| **T8** | 0.591 | *<.0001** | 0.207 | *0.0014** | 0.501 | *<.0001** | 0.145 | *0.1337* | 0.679 | *<.0001** | 0.268 | *0.0037** |
| **T9** | 0.647 | *<.0001** | 0.218 | *0.0002** | 0.558 | *<.0001** | 0.143 | *0.1637* | 0.735 | *<.0001** | 0.294 | *0.0004** |
| **T10** | 0.681 | *<.0001** | 0.257 | *<.0001** | 0.594 | *<.0001** | 0.189 | *0.0021** | 0.767 | *<.0001** | 0.324 | *<.0001** |
| **T11** | 0.707 | *<.0001** | 0.276 | *<.0001** | 0.604 | *<.0001** | 0.190 | *0.0019** | 0.809 | *<.0001** | 0.362 | *<.0001** |
| **T12** | 0.718 | *<.0001** | 0.303 | *<.0001** | 0.634 | *<.0001** | 0.221 | *<.0001* | 0.800 | *<.0001** | 0.386 | *<.0001** |
| **T13** | 0.710 | *<.0001** | 0.286 | *<.0001** | 0.615 | *<.0001** | 0.199 | *0.0006** | 0.803 | *<.0001** | 0.373 | *<.0001** |

* indicates within-arm significance after Bonferroni correction (α=.05/12)

**Table S9.** Mean scores in knowledge that condoms are effective at preventing HIV and *P*-values for difference from baseline mean score by study arm for all participants.

|  | **All participants** | | | | **Female participants** | | | | **Male participants** | | | |
| --- | --- | --- | --- | --- | --- | --- | --- | --- | --- | --- | --- | --- |
|  | **Intervention** | | **Control** | | **Intervention** | | **Control** | | **Intervention** | | **Control** | |
|  | Mean score | *P-value* | Mean score | *P-value* | Mean score | *P-value* | Mean score | *P-value* | Mean score | *P-value* | Mean score | *P-*value |
| **T1** | 0.591 |  | 0.617 |  | 0.543 |  | 0.604 |  | 0.638 |  | 0.630 |  |
| **T2** | 0.716 | *<.0001** | 0.646 | *0.1413* | 0.683 | *<.0001** | 0.627 | *0.4035* | 0.749 | *0.0003** | 0.665 | *0.2082* |
| **T3** | 0.706 | *<.0001** | 0.636 | *0.341* | 0.673 | *<.0001** | 0.623 | *0.5033* | 0.739 | *0.0044* | 0.651 | *0.4883* |
| **T4** | 0.709 | *<.0001** | 0.670 | *0.0124* | 0.697 | *<.0001** | 0.626 | *0.4235* | 0.720 | *0.0199* | 0.714 | *0.0086* |
| **T5** | 0.680 | *0.0005** | 0.655 | *0.0874* | 0.643 | *0.0064* | 0.610 | *0.8479* | 0.717 | *0.0301* | 0.701 | *0.0304* |
| **T6** | 0.675 | *0.0011** | 0.670 | *0.03* | 0.605 | *0.0944* | 0.612 | *0.8039* | 0.744 | *0.0033** | 0.728 | *0.006* |
| **T7** | 0.711 | *<.0001** | 0.645 | *0.2288* | 0.652 | *0.0039** | 0.590 | *0.6611* | 0.768 | *0.0005** | 0.702 | *0.0383* |
| **T8** | 0.688 | *0.0002** | 0.660 | *0.0762* | 0.609 | *0.0709* | 0.607 | *0.9323* | 0.766 | *0.0004** | 0.714 | *0.02* |
| **T9** | 0.727 | *<.0001** | 0.669 | *0.0341* | 0.659 | *0.0022** | 0.622 | *0.5619* | 0.795 | *<.0001** | 0.715 | *0.0211* |
| **T10** | 0.738 | *<.0001** | 0.677 | *0.0182* | 0.658 | *0.0026** | 0.613 | *0.8038* | 0.817 | *<.0001** | 0.742 | *0.0025** |
| **T11** | 0.737 | *<.0001** | 0.679 | *0.011* | 0.668 | *0.0005** | 0.618 | *0.6661* | 0.805 | *<.0001** | 0.740 | *0.0018** |
| **T12** | 0.738 | *<.0001** | 0.671 | *0.0337* | 0.673 | *0.0007** | 0.606 | *0.9465* | 0.802 | *<.0001** | 0.737 | *0.0038** |
| **T13** | 0.748 | *<.0001** | 0.664 | *0.0618* | 0.690 | *0.0001** | 0.593 | *0.7492* | 0.806 | *<.0001** | 0.736 | *0.0041** |

* indicates within-arm significance after Bonferroni correction (α=.05/12)

**Table S10:** Cross-arm difference in mean change in behavioral determinants relative to baseline and associated *P*-values at four timepoints for supplemental outcomes.

| **Timepoint** | | **T2** | | **T7** | | **T9** | | **T13** | |
| --- | --- | --- | --- | --- | --- | --- | --- | --- | --- |
| **Mean age (years)** | | 14.1 | | 15.6 | | 16.4 | | 17.7 | |
|  | | Difference in change in mean score | *P-value* | Difference in change in mean score | *P-value* | Difference in change in mean score | *P-value* | Difference in change in mean score | *P-value* |
| **BEHAVIORAL INTENTIONS** | | | | | | | | | |
| ***Intention to talk to a partner about pregnancy prevention*** | | | | | | | | | |
|  | All | 0.107 | *<.0001^†^* | 0.077 | *0.0043^†^* | 0.057 | *0.0317* | 0.056 | *0.0370* |
|  | Female | 0.117 | *.0014^†^* | 0.071 | *.0531* | 0.048 | *.1914* | 0.065 | *.0800* |
|  | Male | 0.098 | *.0092^†^* | 0.083 | *.036* | 0.067 | *.0855* | 0.047 | *.2241* |
| **SELF-EFFICACY** | | | | | | | | | |
| ***Self-efficacy to talk to a partner about HIV prevention*** | | | | | | | | | |
|  | All | 0.085 | *<.0001^†^* | 0.053 | *.0067^†^* | 0.059 | *.0031^†^* | 0.040 | *.0407* |
|  | Female | 0.069 | *.0097^†^* | 0.023 | *.4024* | 0.031 | *.2678* | 0.027 | *.3107* |
|  | Male | 0.101 | *.0005^†^* | 0.083 | *.0028^†^* | 0.088 | *.0022^†^* | 0.052 | *.0591* |
| ***Self-efficacy to talk to a partner about pregnancy prevention*** | | | | | |  | |  | |
|  | All | 0.083 | *.0003^†^* | 0.051 | *.0213* | 0.044 | *.0522* | 0.037 | *.1033* |
|  | Female | 0.063 | *.0484* | 0.023 | *.4687* | 0.020 | *.5185* | 0.016 | *.6041* |
|  | Male | 0.103 | *.0018^†^* | 0.080 | *.0106^†^* | 0.068 | *.0399* | 0.058 | *.0786* |
| **KNOWLEDGE** | | | | | | | | | |
| ***Knowledge that condoms are effective for pregnancy prevention*** | | | | | | | | | |
|  | All | 0.128 | *<.0001^†^* | 0.071 | *.0325* | 0.058 | *.0950* | 0.062 | *.0814* |
|  | Female | 0.132 | *.0007^†^* | 0.071 | *.1136* | 0.046 | *.3456* | 0.047 | *.3418* |
|  | Male | 0.123 | *.0020^†^* | 0.069 | *.1526* | 0.068 | *.1592* | 0.076 | *.1367* |
