# Supplementary material for: Longitudinal Effects of a Smartphone Game (Tumaini) for HIV Prevention Among Kenyan Adolescents: 45-Month Trajectories of Condom Use–Related Proximal Outcomes From a Randomized Controlled Trial
Source: J Med Internet Res. 2026 Mar 10;28:e83982. doi: 10.2196/83982 (PMC13014075; doi:10.2196/83982)

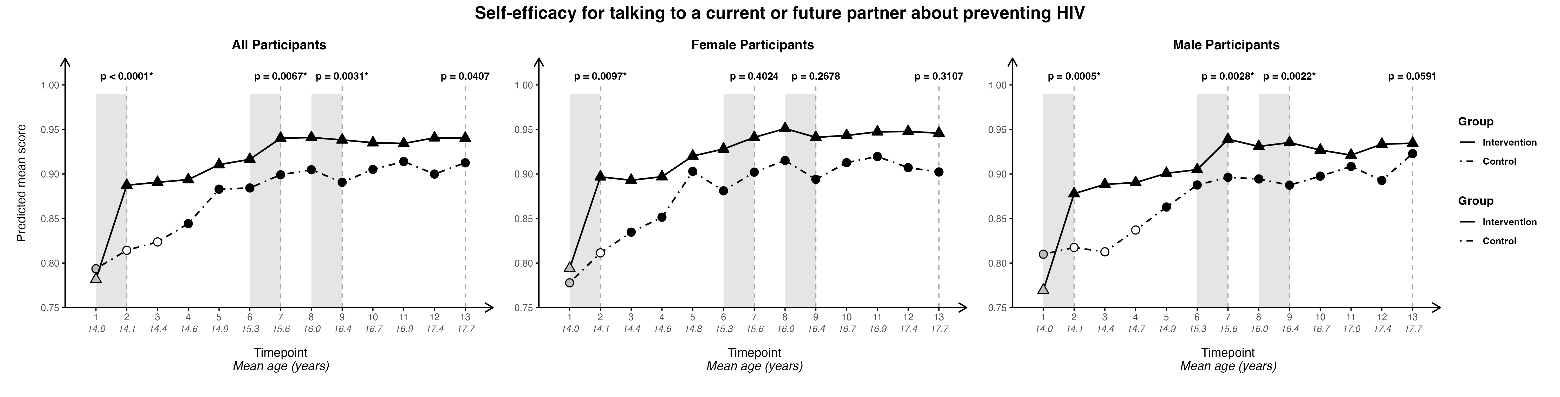


**Figure S1: Self-efficacy to talk to a partner about HIV prevention**


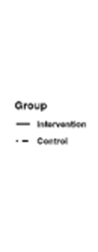

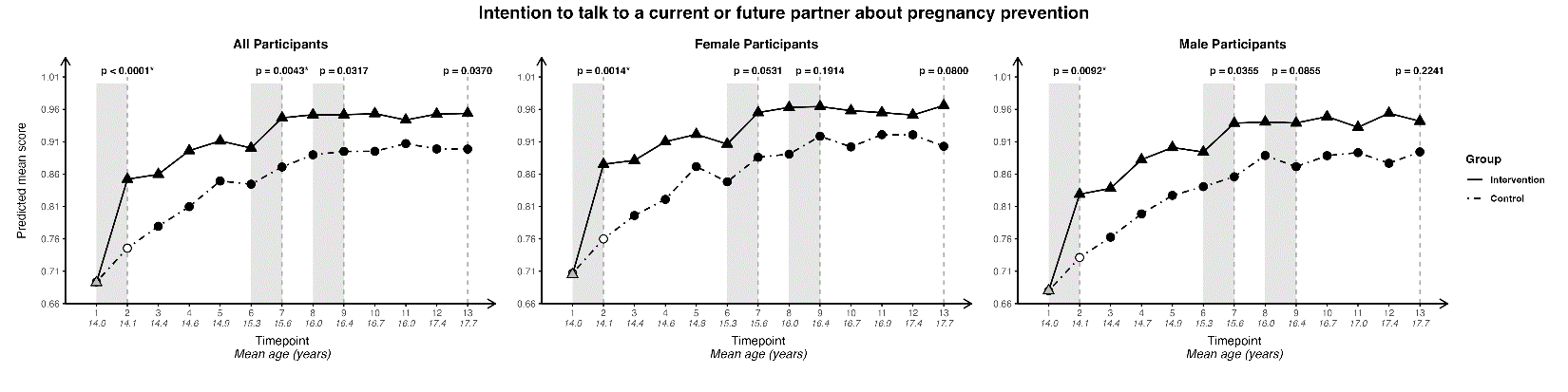


**Figure S2: Intention to talk to a partner about pregnancy prevention**


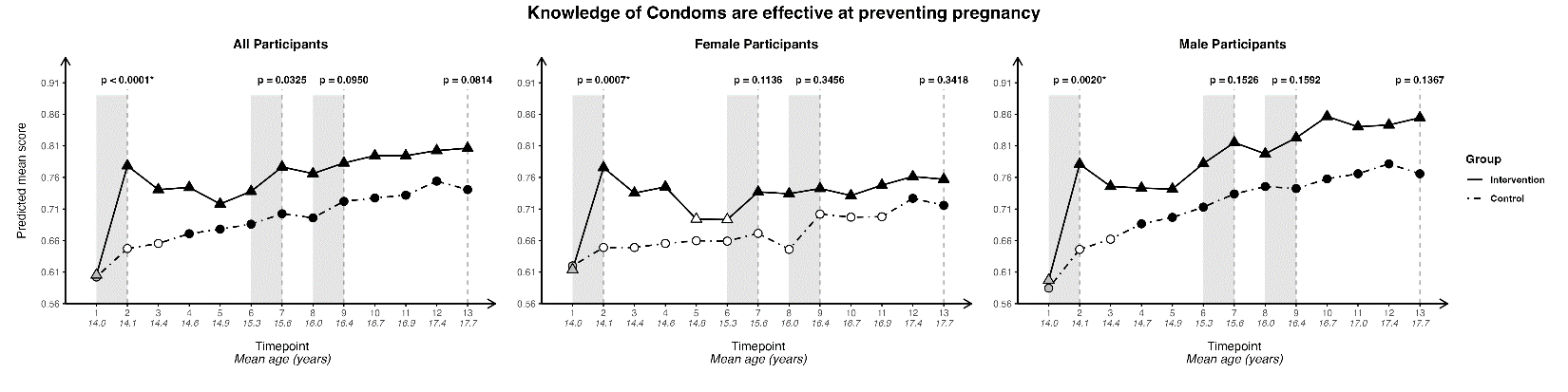


**Figure S4: Knowledge that condoms are an effective way to prevent pregnancy**


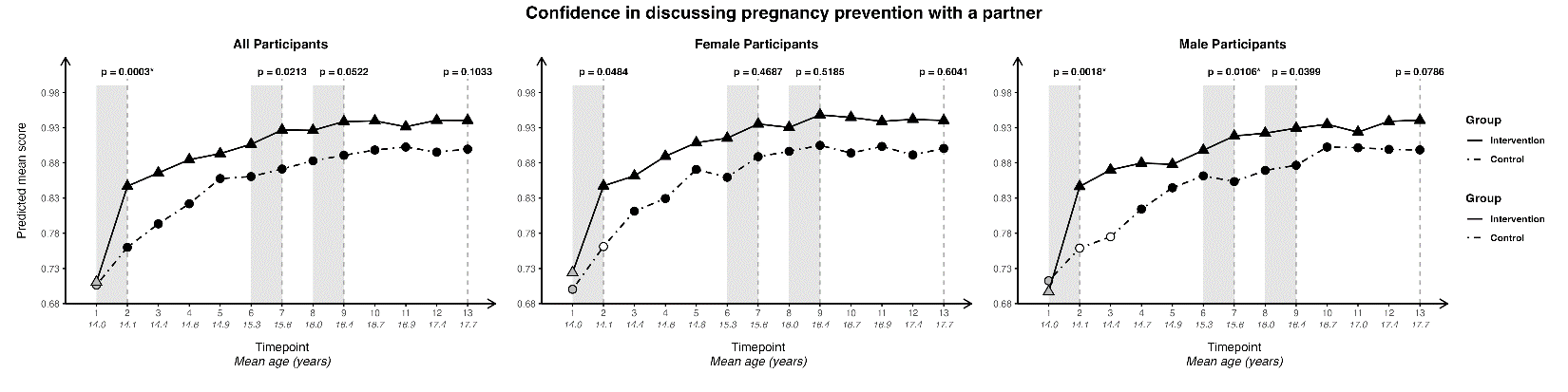


**Figure S3: Self-efficacy to discuss pregnancy prevention with a partner**


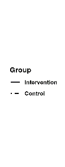

Supplement: Multimedia Appendix 2 [file jmir_v28i1e83982_app2.docx]
