# Supplementary figures and images for: Longitudinal Effects of a Smartphone Game (Tumaini) for HIV Prevention Among Kenyan Adolescents: 45-Month Trajectories of Condom Use–Related Proximal Outcomes From a Randomized Controlled Trial
Source: J Med Internet Res. 2026 Mar 10;28:e83982. doi: 10.2196/83982 (PMC13014075; doi:10.2196/83982)

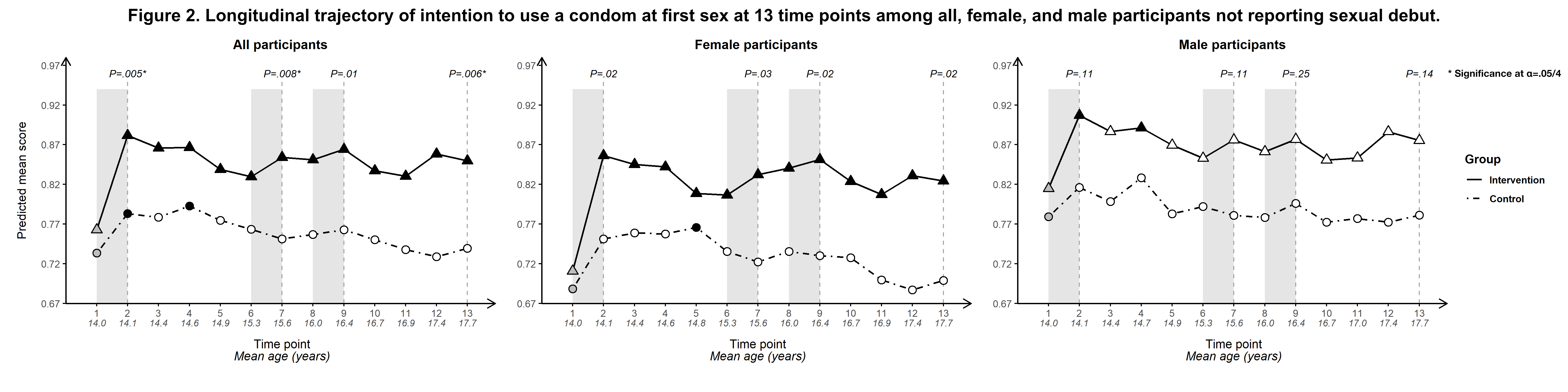

Supplement: Multimedia Appendix 4 [file jmir_v28i1e83982_app4.zip › Higher Resolution Figures 2-9/Figure 2 High Resolution.png]

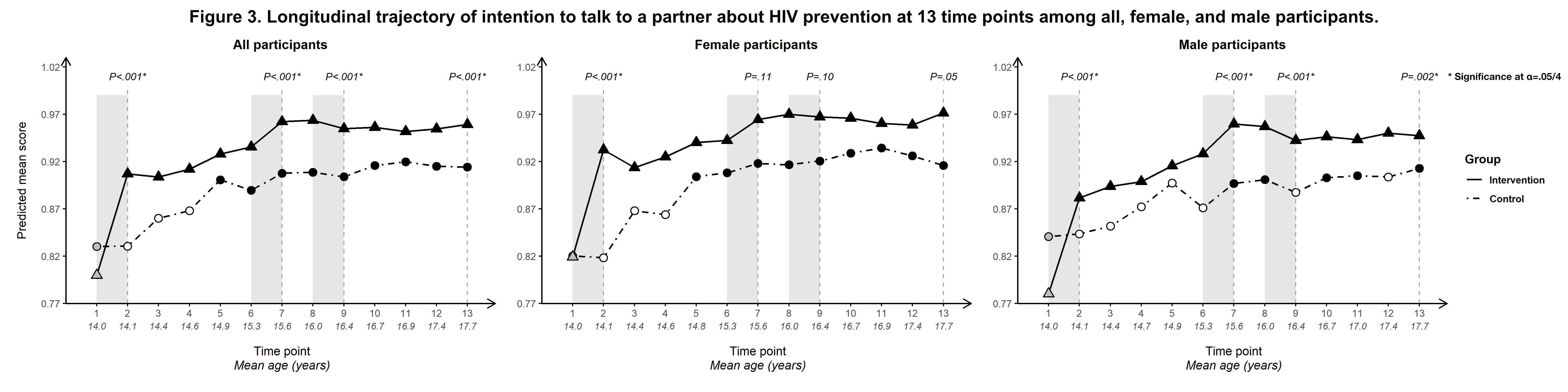

Supplement: Multimedia Appendix 4 [file jmir_v28i1e83982_app4.zip › Higher Resolution Figures 2-9/Figure 3 High Resolution.png]

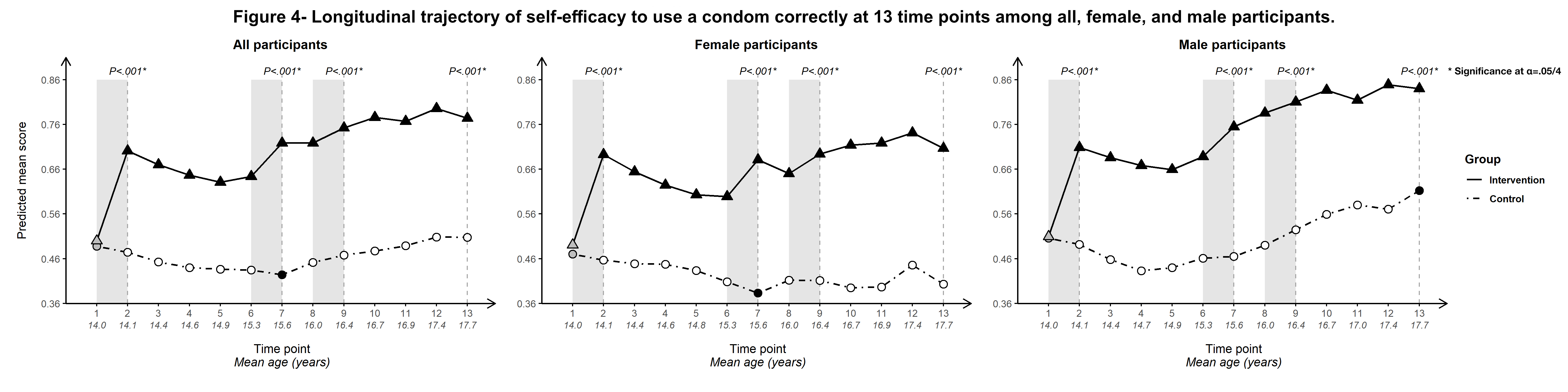

Supplement: Multimedia Appendix 4 [file jmir_v28i1e83982_app4.zip › Higher Resolution Figures 2-9/Figure 4 High Resolution.png]

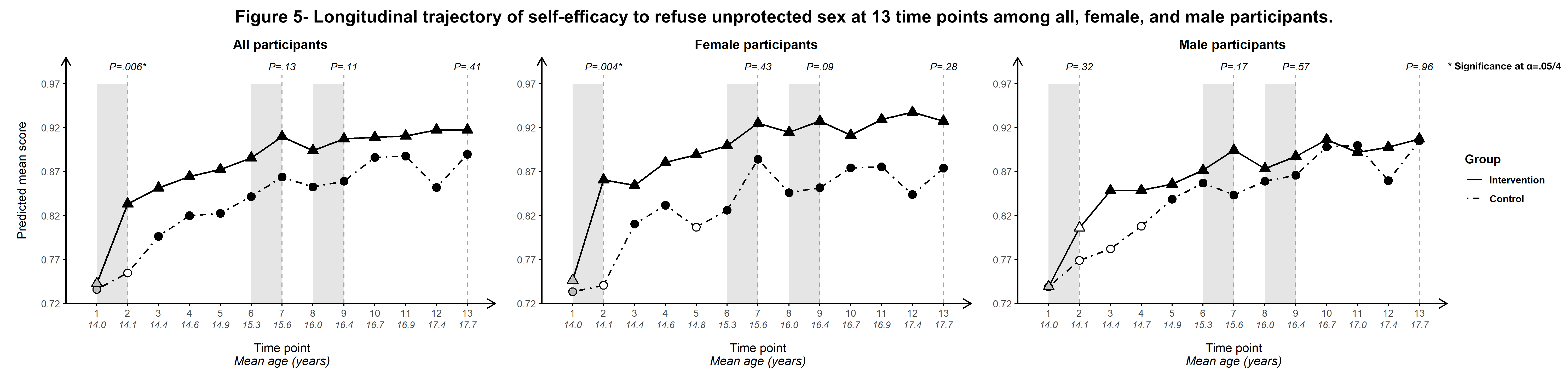

Supplement: Multimedia Appendix 4 [file jmir_v28i1e83982_app4.zip › Higher Resolution Figures 2-9/Figure 5 High Resolution.png]

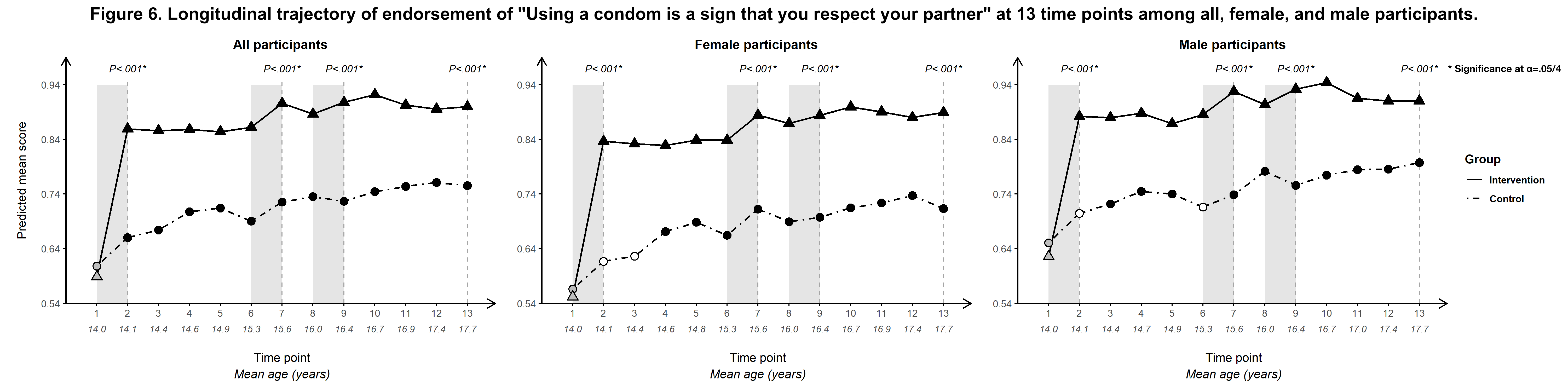

Supplement: Multimedia Appendix 4 [file jmir_v28i1e83982_app4.zip › Higher Resolution Figures 2-9/Figure 6 High Resolution.png]

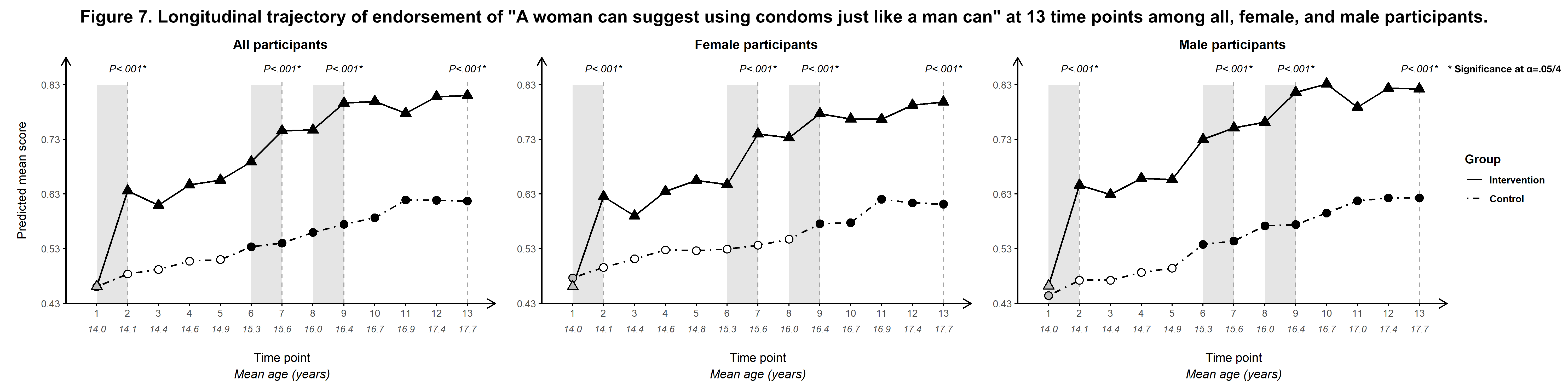

Supplement: Multimedia Appendix 4 [file jmir_v28i1e83982_app4.zip › Higher Resolution Figures 2-9/Figure 7 High Resolution.png]

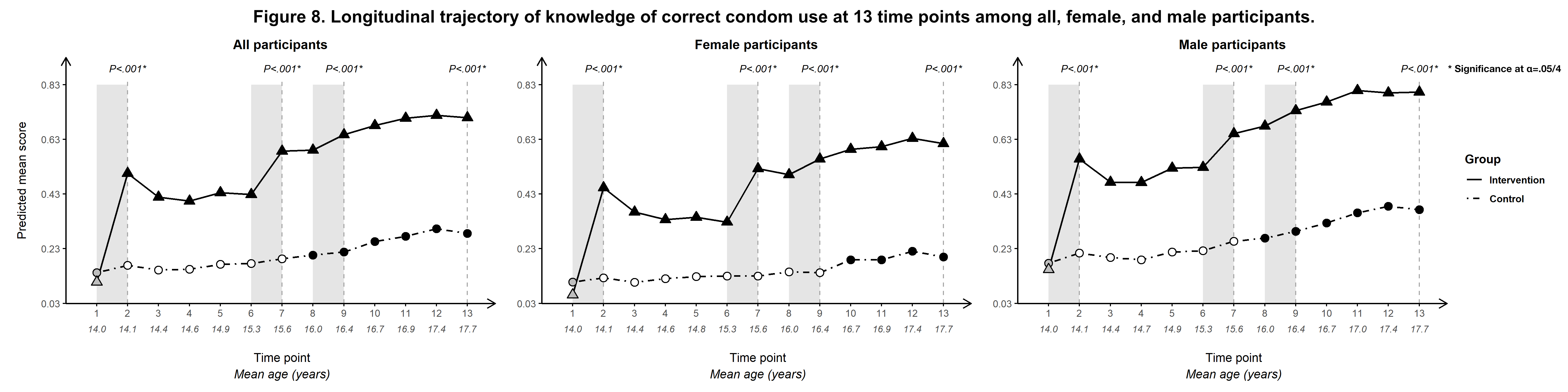

Supplement: Multimedia Appendix 4 [file jmir_v28i1e83982_app4.zip › Higher Resolution Figures 2-9/Figure 8 High Resolution.png]

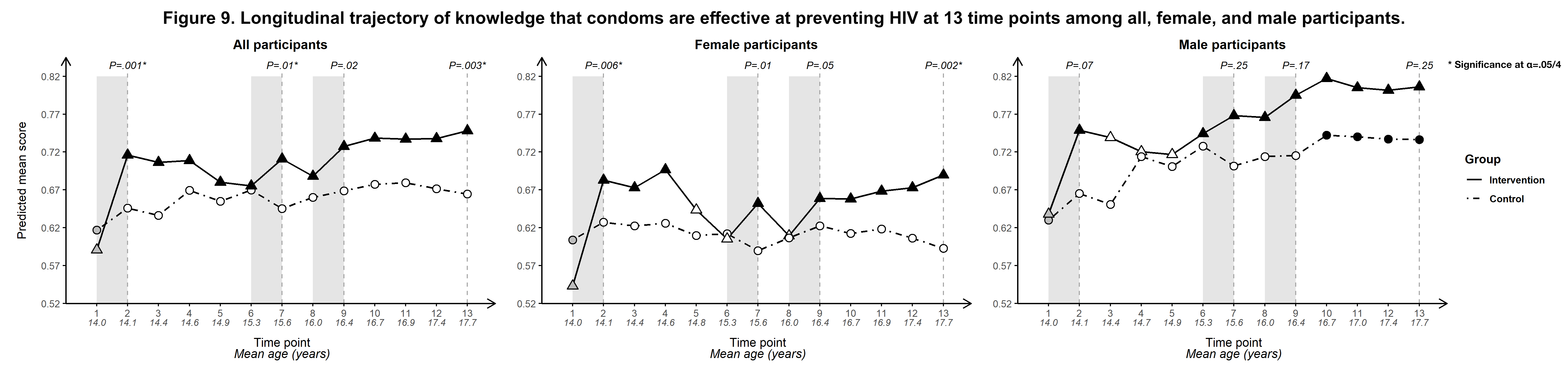

Supplement: Multimedia Appendix 4 [file jmir_v28i1e83982_app4.zip › Higher Resolution Figures 2-9/Figure 9 High Resolution.png]
